# Development of a *Staphylococcus aureus* reporter strain with click beetle red luciferase for enhanced *in vivo* imaging of experimental bacteremia and mixed infections

**DOI:** 10.1038/s41598-019-52982-0

**Published:** 2019-11-13

**Authors:** Robert J. Miller, Heidi A. Crosby, Katrin Schilcher, Yu Wang, Roger V. Ortines, Momina Mazhar, Dustin A. Dikeman, Bret L. Pinsker, Isabelle D. Brown, Daniel P. Joyce, Jeffrey Zhang, Nathan K. Archer, Haiyun Liu, Martin P. Alphonse, Julie Czupryna, William R. Anderson, Nicholas M. Bernthal, Lea Fortuno-Miranda, Jeff W. M. Bulte, Kevin P. Francis, Alexander R. Horswill, Lloyd S. Miller

**Affiliations:** 1 Department of Dermatology, Johns Hopkins University School of Medicine, Baltimore, Maryland USA; 2 Department of Immunology & Microbiology, University of Colorado Anschutz Medical Campus, Aurora, Colorado 80045 USA; 3 PerkinElmer, Hopkinton, Massachusetts USA; 4 Department of Orthopaedic Surgery, David Geffen School of Medicine at UCLA, Santa Monica, California USA; 5 Russell H. Morgan Department of Radiology and Radiological Science, Division of MR Research, Johns Hopkins University School of Medicine, Baltimore, Maryland 21205 USA; 6 Cellular Imaging Section and Vascular Biology Program, Institute for Cell Engineering, Johns Hopkins University School of Medicine, Baltimore, Maryland 21205 USA; 7 Department of Chemical & Biomolecular Engineering, Johns Hopkins University Whiting School of Engineering, Baltimore, Maryland 21205 USA; 8 Department of Oncology, Johns Hopkins University School of Medicine, Baltimore, Maryland 21205 USA; 9 Department of Biomedical Engineering, Johns Hopkins University School of Medicine, Baltimore, Maryland 21205 USA; 10 Denver VA Healthcare System, Denver, Colorado USA; 11 Department of Medicine, Division of Infectious Diseases, Johns Hopkins University School of Medicine, Baltimore, Maryland 21287 USA; 12 Department of Orthopaedic Surgery, Johns Hopkins University School of Medicine, Baltimore, Maryland 21287 USA; 13 Department of Materials Science and Engineering, Johns Hopkins University, Baltimore, Maryland 21218 USA

**Keywords:** Bacterial techniques and applications, Pathogens, Bacterial infection, Preclinical research

## Abstract

*In vivo* bioluminescence imaging has been used to monitor *Staphylococcus aureus* infections in preclinical models by employing bacterial reporter strains possessing a modified *lux* operon from *Photorhabdus luminescens*. However, the relatively short emission wavelength of *lux* (peak 490 nm) has limited tissue penetration. To overcome this limitation, the gene for the click beetle (*Pyrophorus plagiophtalamus*) red luciferase (*luc*) (with a longer >600 emission wavelength), was introduced singly and in combination with the *lux* operon into a methicillin-resistant *S. aureus* strain. After administration of the substrate D-luciferin, the *luc* bioluminescent signal was substantially greater than the *lux* signal *in vitro*. The *luc* signal had enhanced tissue penetration and improved anatomical co-registration with infected internal organs compared with the *lux* signal in a mouse model of *S. aureus* bacteremia with a sensitivity of approximately 3 × 10^4^ CFU from the kidneys. Finally, in an *in vivo* mixed bacterial wound infection mouse model, *S. aureus luc* signals could be spectrally unmixed from *Pseudomonas aeruginosa lux* signals to noninvasively monitor the bacterial burden of both strains. Therefore, the *S. aureus luc* reporter may provide a technological advance for monitoring invasive organ dissemination during *S. aureus* bacteremia and for studying bacterial dynamics during mixed infections.

## Introduction

*Staphylococcus aureus* is a major human pathogen that causes the majority of skin infections as well as invasive and life-threatening infections such as bacteremia, pneumonia, surgical site infections and organ abscesses^1,2^. *S. aureus* bacteremia is particularly problematic as the mortality rate has remained between 14 to 29% despite the use of antibiotics with coverage against antibiotic-resistant strains (such as methicillin-resistant *S. aureus* [MRSA]) and advances in supportive measures^3–6^. To study the pathogenesis of *S. aureus* infections in preclinical animal models, *in vivo* whole animal bioluminescence imaging (BLI) has been used with bioluminescent *S. aureus* strains expressing the *luxABCDE* (*lux*) operon, adapted from the bacterial insect pathogen *Photorhabdus luminescens*^7–10^. For *S. aureus*, Gram-positive ribosomal binding sites have been introduced upstream of each of *lux* gene, resulting in the endogenous emission of bioluminescent light from live and actively metabolizing *S. aureus* bacteria^11–15^. A strong promoter that is active in all bacterial growth phases can be inserted upstream of the *lux* genes for improved light production^13,14^. Furthermore, if the *lux* operon construct is stably integrated into the bacterial chromosome or into a stable plasmid (rather than an unstable antibiotic selection plasmid^13,16^), light production is maintained in all progeny and the BLI signals highly correlate with *ex vivo* colony forming units (CFU)^13,17–21^. The use of *in vivo* BLI with bioluminescent *S. aureus* strains has permitted the noninvasive and longitudinal monitoring of the bacterial burden, which has provided key information about the infectious course and disease pathogenesis in skin and soft tissue infections^13,17,22–26^ as well as musculoskeletal infections^16,19,27–34^. In addition, this technology has been used to evaluate therapeutics, such as antibiotics^18,20,35–39^, active and passive vaccines^29,40,41^ and other antimicrobials^37,42^ as well as *S. aureus*-specific diagnostic imaging probes^27,43,44^.

Although *in vivo* BLI in conjunction with *S. aureus* bioluminescent strains has been used in many preclinical models of infection, the light emitted from the *S. aureus lux* reporter strains has a relatively short wavelength (peak = 490 nm^45^), which limits light penetration through deeper tissues^7,8^. Therefore, in deep-seated and invasive *S. aureus* infections, the emitted *in vivo* BLI signal is quenched by the surrounding tissue and no longer accurate as it underestimates the actual *in vivo* bacterial burden^7,8^. In addition, the light production by the *S. aureus lux* reporter strains is also limited by the metabolic activity of the bacteria and it is often difficult to detect dim signals from metabolically inactive bacteria such as bacteria present in biofilms^38,46^. Taken together, existing *in vivo* BLI approaches with *S. aureus lux* strains are more accurate in monitoring the *in vivo* bacterial burden for more superficial *S. aureus* infections such as skin and musculoskeletal infections, but its use in invasive infections is limited.

In the present study, we set out to improve the capability and accuracy of detecting BLI signals in invasive *S. aureus* infections. First, we further modified the *lux* operon for improved endogenous light production in a new bioluminescent *S. aureus* strain. Second, since the click beetle (*Pyrophorus plagiophtalamus*) red luciferase (*luc*) has a substantially longer emission wavelength of light (peak >600 nm) after exogenous substrate exposure^47–51^, we reasoned that a *S. aureus luc* reporter strain might result in better tissue penetration than a *S. aureus lux* reporter strain. Therefore, we also developed a *luc* construct that was introduced singly or along with the *lux* construct into a *S. aureus* strain to develop new *lux*, *luc* and dual *lux* + *luc* reporter *S. aureus* strains. The bioluminescent signals from these *lux* and *luc* expressing *S. aureus* strains were then evaluated *in vitro* and in invasive (*i.e*., bacteremia) and more superficial (*i.e*., skin and musculoskeletal) preclinical models of *S. aureus* infection *in vivo*.

## Results

### Generation of a new lux expressing *S. aureus* strain

**Figure 1:**
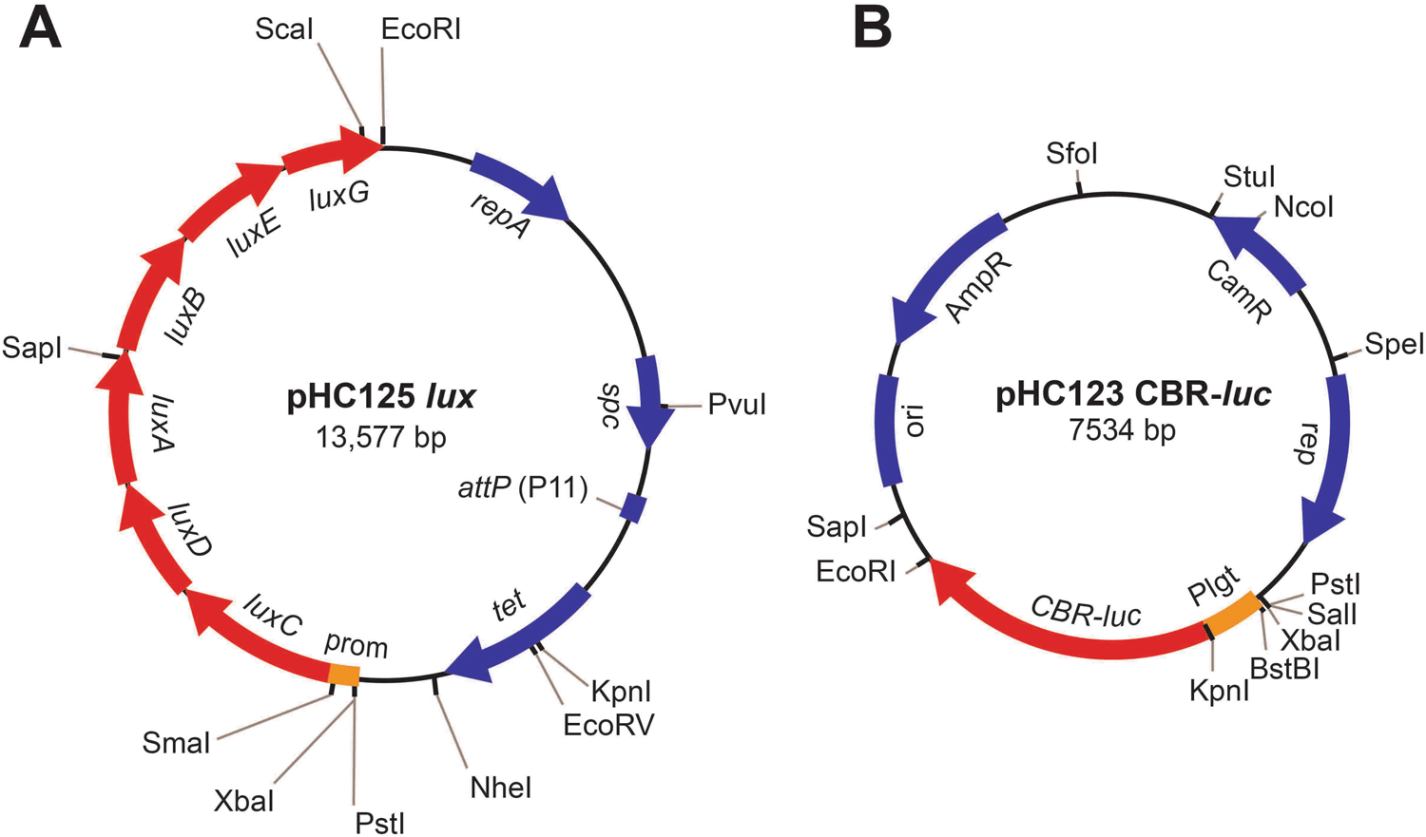
Genetic maps of *lux* and *luc* plasmid constructs. (**A**) Plasmid pHC125 *lux* used to generate chromosomally integrated *luxCDABEG* construct. Expression is driven by the P_CP25_ and P_CAP_ promoters (prom). Plasmid stably integrates at the phage 11 attachment site in *S. aureus*. (**B**) Plasmid pHC123 CBR-*luc* carrying constitutively expressed click beetle red luciferase (CBR-*luc*). Expression is driven by the P_lgt_ promoter.

To generate an improved bioluminescent *lux* expressing *S. aureus* strain, the gene sequence *luxCDABEG* derived from the bioluminescent bacterial insect pathogen *Photorhabdus luminescens* was synthesized with Gram-positive ribosome binding sites at the start sites of each respective *lux* gene. This cassette has two strong promoters at the start called P_CP25_ and P_CAP_, followed by an excisable stem loop transcriptional terminator. Expression of the *lux* genes is driven by readthrough from these strong promoters. This complete *lux* cassette was cloned into plasmid pLL29^52^ to generate plasmid pHC125 *lux* (Fig. 1A). This plasmid was integrated at the ϕ11 attachment site on the chromosome of the CA-MRSA LAC strain AH1263^53^ to generate the new bioluminescent strain AH4807 (*lux*). The construct is stable without selection and easily moved by bacteriophage transduction to other *S. aureus* strains such as CA-MRSA USA400 MW2^54^ to generate strain AH4821 (*lux*) and MSSA Newman^55^ to generate strain AH5016 (*lux*).

We compared the new AH4807 (*lux*) against the existing bioluminescent *S. aureus* strains USA300 LAC::*lux*^15^, LAC4303 (*lux*)^14,56^ and Xen36 (*lux*)^11^ (Table 1) in terms of growth, luminescence output, and stability *in vitro*. For these comparison strains, USA300 LAC::*lux* was constructed by moving the original *lux* kanamycin resistant (Kan^R^) cassette from strain Xen29^57^. LAC4303 (*lux*) has the *lux* construct on an integrated plasmid on the bacterial chromosome of USA300 LAC, and Xen36 (*lux*) has the *lux* construct inserted on a stable plasmid in the methicillin-sensitive *S. aureus* strain Wright. All four strains grow fairly similarly, with USA300 LAC::*lux* lagging slightly behind (see Supplemental Data, Fig. S1A). LAC4303 (*lux*) generated the most bioluminescence during a 10-hour time course, followed by both AH4807 (*lux*) and Xen36 (*lux*) which behaved similarly over time (Fig. S1A), and finally USA300 LAC::*lux* had the lowest luminescence output. LAC4303 (*lux*) is constructed with a temperature-sensitive plasmid called pRP1195^14^ and we predicted this plasmid might undergo excision from the chromosome at lower temperatures. Indeed, we observed significant excision rates at 30 °C as determined by PCR (Fig. S1B,C), which were reduced at 37 °C and non-existent at 43 °C. Despite the instability, we did not observe a deleterious impact on overall LAC4303 (*lux*) luminescence at lower temperatures. The AH4807 (*lux*) plasmid pHC125 was stable at all temperatures as anticipated (Fig. S1B,C).

**Table 1:** *S. aureus* strains.

Name	Genotype	Features	Reference
AH4775 (*luc*)	LAC + pHC123 (CBR-*luc*, Cam^R^)	CBR-*luc*	This paper
AH4807 (*lux*)	LAC int::*luxCDABEG*	*lux*	This paper
AH4826 (*lux* + *luc*)	LAC int::*luxCDABEG* pHC123 (CBR-*luc*, Cam^R^)	*lux* and CBR-*luc*	This paper
USA300 LAC::*lux*	LAC int::*luxABCDE*	*lux*	^15^
LAC4303 (*lux*)	LAC strain JE2 int::*luxBADCE*	*lux*	^56^
Xen36 (*lux*)	Wright (ATCC 49525) + *luxABCDE* (on a native plasmid)	*lux*	^11^
AH4821 (*lux*)	MW2 int::*luxCDABEG*	*lux*	This paper
AH5016 (*lux*)	Newman int::*luxCDABEG*	*lux*	This paper
AH5556 (*luc*)	Newman + pHC123 (CBR-*luc*, Cam^R^)	CBR-*luc*	This paper
AH5557 (*luc*)	MW2 + pHC123 (CBR-*luc*, Cam^R^)	CBR-*luc*	This paper
AH5558 (*lux* + *luc*)	Newman int::*luxCDABEG* pHC123 (CBR-*luc*, Cam^R^)	*lux* and CBR-*luc*	This paper
AH5559 (*lux* + *luc*)	MW2 int::*luxCDABEG* pHC123 (CBR-*luc*, Cam^R^)	*lux* and CBR-*luc*	This paper

Cam^R^ = chloramphenicol resistant.

### Generation of *luc* and *lux* + *luc* expressing *S. aureus* strains

Since the click beetle (*Pyrophorus plagiophtalamus*) red (CBR) luciferase (*luc*) has a longer emission wavelength of light (peak >600 nm^47–51^) than *lux* (peak 490 nm^45^) (Table 2), we generated *luc* and *lux* + *luc* expressing CA-MRSA strains. The CBR *luc* gene was synthesized and cloned into the *S. aureus* shuttle vector pCM28^53^ under the control of the *hprK*/*lgt* constitutive promoter, generating plasmid pHC123 CBR-*luc* (Fig. 1B). This plasmid was then transduced into the CA-MRSA LAC strain to generate the *luc* expressing strain AH4775 (*luc*) and into AH4807 (*lux*) to generate the *lux* + *luc* expressing strain AH4826 (*lux* + *luc*). In addition, the *luc *construct was also transduced in two divergent *S. aureus* strains, USA400 MW2 to generate AH5557 (*luc*) and AH5559 (*lux* + *luc*) and MSSA Newman to generate AH5556 (*luc*) and AH5558 (*lux* + *luc*).

**Table 2:** Peak wavelengths (nm) of *lux* versus CBR-*luc* + D-Luciferin substrate.

	Peak	Reference
*Lux* (*Photorhabdus luminescens*)	490	^45^
*Luc* (CBR) + D-Luciferin	614	^58^

### Bacterial growth and light production of *lux*, *luc* and *lux* + *luc S. aureus* strains *in vitro*

**Figure 2:**
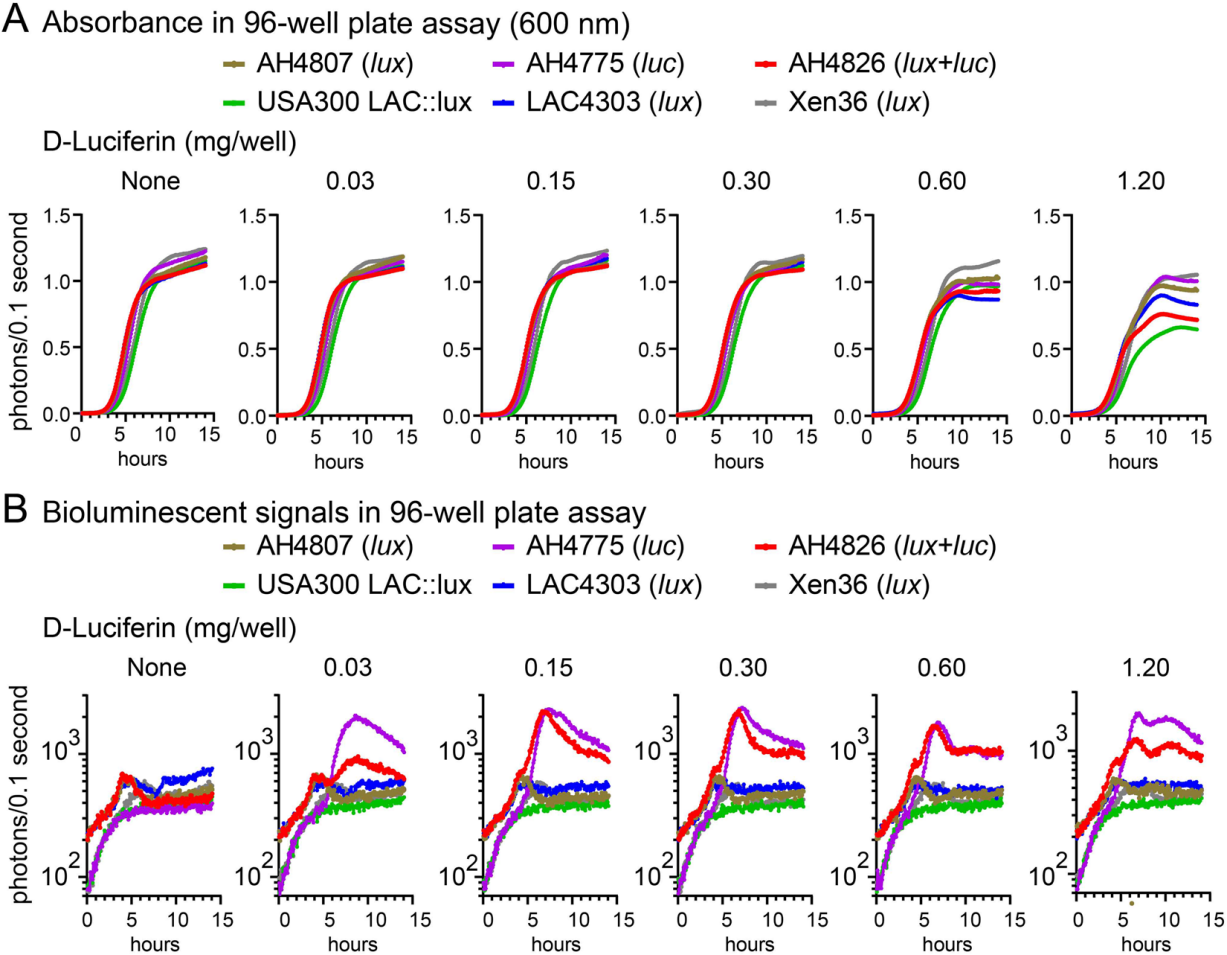
*In vitro* bacterial growth curves and bioluminescence signals. AH4807 (*lux*), AH4775 (*luc*), AH4826 (*lux* + *luc*), USA300 LAC::*lux*, LAC4303 (*lux*) and Xen36 (*lux*) (all 1 × 10^4^ CFU) were cultured in 96-well plates without (none) and with the addition of different concentrations (0.03–1.2 mg/240 µL TSB/well) of D-Luciferin for 0–14 hours and absorbance at 600 nm (A_600_) (**A**) and bioluminescent signals (photons/0.1 second acquisition time) (**B**) were measured every 5 minutes on a plate reader (BioTek H1 Synergy) (n = 4 replicates with 2 iterations).

Next, *in vitro* assays were performed to compare AH4807 (*lux*), AH4775 (*luc*) and AH4826 (*lux* + *luc*) against the existing bioluminescent *S. aureus* strains USA300 LAC::*lux*, LAC4303 (*lux*) and Xen36 (*lux*) (Table 1). The *lux* expressing strains naturally emit light due to endogenous substrates produced during bacterial metabolism^7–10^ whereas *luc* expressing strains produce light only in the presence of an exogenous substrate, firefly D-Luciferin^47–51^. Therefore, the *in vitro* assays were conducted without an exogenous substrate to detect *lux* signals and in the presence of D-Luciferin (0.03–1.2 mg) to detect *luc* signals. To evaluate for any differences in bacterial growth, all strains were cultured in shaking broth in parallel wells in 96-well plates for 14 hours and absorbance at 600 nm (A_600_) (Fig. 2A) and bioluminescent signals (Fig. 2B) were measured. LAC4303 (*lux*) was used as a comparison strain as it emits brighter bioluminescent signals than other existing *lux* strains (USA300 LAC::*lux* and Xen36 (*lux*)). In the presence of 0.03, 0.15 or 0.30 mg of D-Luciferin, there was no difference in absorbance among all of the strains. However, in the presence of 0.60 mg D-Luciferin, from 10–14 hours there was higher absorbance of the *lux* expressing strains AH4807 and Xen36 (*lux*) than LAC4303 (*lux*) (*P* < 0.05) whereas the absorbance of USA300 LAC::*lux*, AH4775 (*luc*) and AH4826 (*lux* + *luc*) did not differ compared with LAC4303 (*lux*). At 1.20 mg of D-Luciferin, from 7–14 hours Xen36 (*lux*) and AH4775 (*luc*) had higher absorbance than LAC4303 (*lux*) (*P* < 0.05) whereas USA300 LAC::*lux* and AH4826 (*lux* + l*uc*) had lower absorbance) compared with LAC4303 (*lux*).

Irrespective of any effects of high concentrations of D-Luciferin (0.60 and 1.20 mg) on bacterial growth *in vitro*, all concentrations of D-Luciferin tested resulted in markedly increased bioluminescent signals with the *luc* expressing strains AH4775 (*luc*) and AH4826 (*lux* + *luc*) strains beginning at 6 hours, peaking at 7–9 hours and then slightly decreasing compared with the lower bioluminescent signals of LAC4303 (*lux*) (which had similar bioluminescent signals as all of the other *lux* expressing strains). To evaluate the new bioluminescent constructs in other *S. aureus* strains, *lux*, *luc* and *lux* + *luc* were expressed in the MW2 and Newman backgrounds and compared to LAC (see Supplemental Data, Fig. S2A,B).

Taken together, although D-Luciferin at high concentrations had some effects on bacterial growth among the bioluminescent *S. aureus* strains, D-luciferin at all concentrations tested resulted in markedly increased bioluminescent signals only from the *luc S. aureus* strains, indicating bioluminescent signals produced by *luc* were a magnitude greater than those produced by *lux in vitro*.

### Enhanced tissue penetration of *luc* compared with *lux* BLI signals *in vitro*

**Figure 3:**
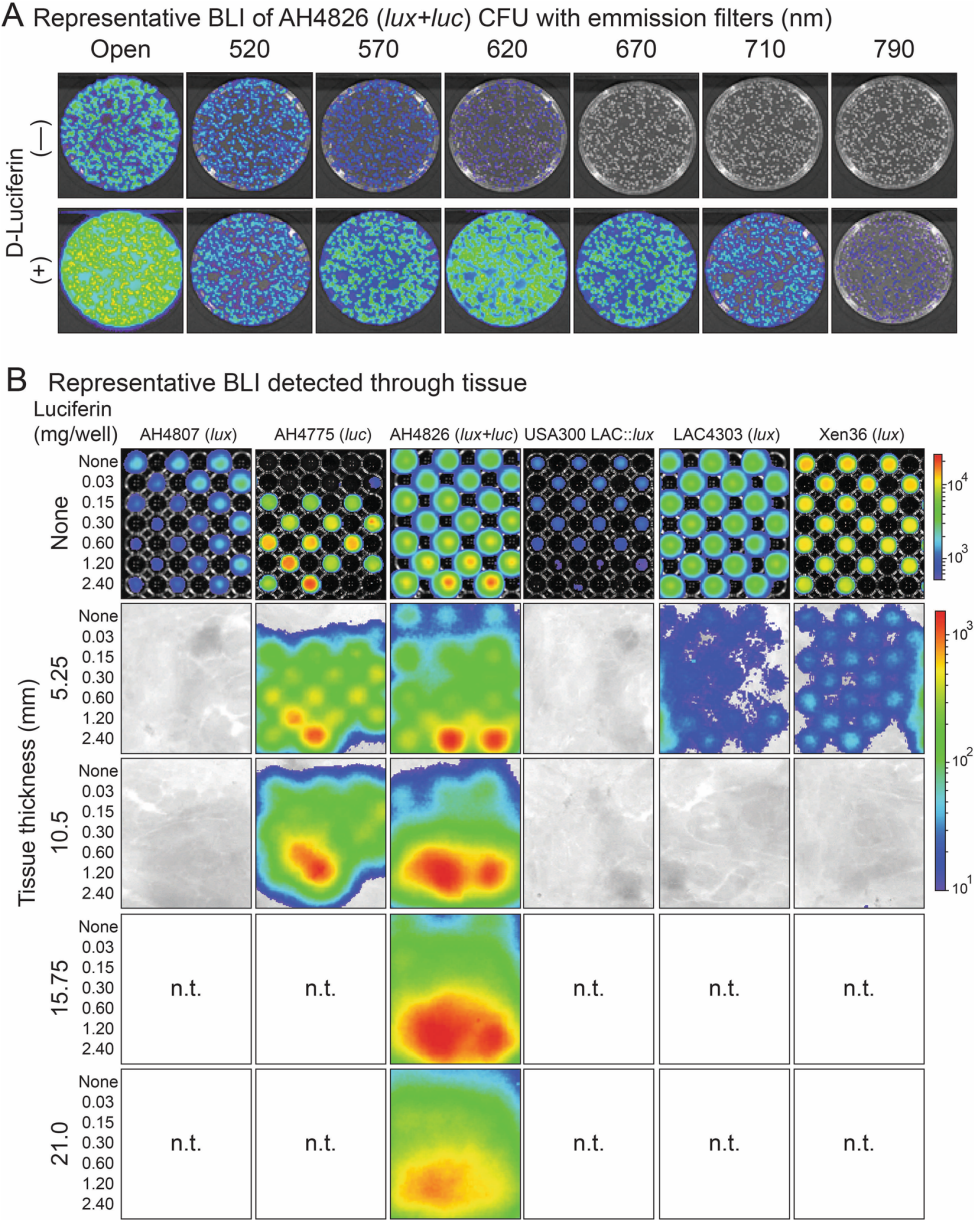
*In vitro lux* versus *luc* BLI signals through tissue. (**A**) AH4826 (*lux* + *luc*) was cultured on bacterial culture petri dishes overnight and BLI was performed for a 30 second acquisition time at 37 °C without (top panels [*lux* signals]) and with the addition of D-Luciferin (600 ng/200 µL PBS pipetted directly onto the surface of the plates) (bottom panels [*lux* + *luc* signals]) on an IVIS Lumina III (PerkinElmer) (n = 3 replicate plates with 2 iterations). Representative BLI signals are provided with no filter (open) and with 520, 570, 620, 670, 710 and 790 nm emission filters. (**B**) AH4807 (*lux*), AH4775 (*luc*), AH4826 (*lux* + *luc*), USA300 LAC::*lux*, LAC4303 (*lux*) and Xen36 (*lux*) (all 1 × 10^9^ CFU) were cultured in 96-well plates (n = 3 replicate wells for each condition with 2 iterations). BLI was performed for a 1 minute acquisition time at 37 °C in an IVIS Lumina III (PerkinElmer) without (none) or with different thicknesses of tissue (sliced cooked ham: none, 5.25, 10.5, 15.75 and 21.0 mm) placed on top of the plate covers ± different concentrations of D-Luciferin (0.03–2.4 mg/280 µL TSB) added to the wells. Representative BLI signals are shown. n.t. = not tested.

To evaluate the BLI signals produced by the *lux* versus *luc* constructs, AH4826 (*lux* + *luc*) was cultured overnight on bacterial culture plates. The plates were then imaged (IVIS Lumina IIII, PerkinElmer) with no filter (open) and with 520, 570, 620, 670, 710 and 790 nm emission filters in the absence (—) (*lux* signals only) or presence (+) (*lux* + *luc* signals) of D-Luciferin added to the surface of the plates (Fig. 3A). With the open filter, D-Luciferin addition to AH4826 (*lux* + *luc*) colonies on the plates resulted in markedly higher BLI signals than in absence of D-Luciferin, suggesting that the light produced by *luc* greatly enhanced the BLI signals of *lux* alone. Furthermore, in the absence of D-Luciferin, the highest BLI signals were detected with the shortest wavelength emission filter (520 nm) and the signals decreased with longer emission filters with no signals detected beyond the 620 nm emission filter. In contrast, in the presence of D-Luciferin, the *lux* + *luc* signals peaked with the 620 nm emission filter and the BLI signals decreased with shorter and longer emission filters away from this peak. Taken together, the BLI signals of AH4826 (*lux* + *luc*) in the absence and presence of D-Luciferin are consistent with the known peak wavelengths of 490 nm for *lux*^45^ and 614 nm for CBR-*luc* in the presence of the D-Luciferin substrate^58^.

Given that CBR-*luc* signals were substantially greater and peaked at a much higher wavelength than those of *lux*, we evaluated whether the addition of D-Luciferin could enhance tissue penetration *in vitro*. This was accomplished by culturing AH4807 (*lux*), AH4775 (*luc*), AH4826 (*lux* + *luc*), USA300 LAC::*lux*, LAC4303 (*lux*) and Xen36 (*lux*) in 96-well plates without (none) or with increasing concentrations of D-Luciferin (0.03–2.4 mg) added to the wells (Fig. 3B). Immediately prior to imaging the plates (IVIS Lumina III), different thicknesses of tissue (sliced cooked ham 5.25 to 21 mm) were placed on top of the plate covers. With no tissue placed on top of the plates, the BLI signals could be detected from all *lux* strains, including AH4826 (*lux* + *luc*) followed by LAC4303 (*lux*), Xen36 (*lux*), AH4807 (*lux*) and USA300 LAC::*lux*. With 5.25 mm of tissue placed on top of the plates, *lux* signals from only AH4826 (*lux* + *luc*), LAC4303 (*lux*) and Xen36 (*lux*) could be detected, and with 10.5 mm of tissue placed on top of the plates, no *lux* signals could be detected. With increasing concentrations of D-Luciferin added, both *luc* expressing strains AH4775 (*luc*) and AH4826 (*luc* + *lux*) had detectable BLI signals through 10.5 mm of tissue. Remarkably, with increasing concentrations of D-Luciferin, AH4826 (*lux* + *luc*) had BLI signals that could be detected through 15.75 and 21.0 mm of tissue. Taken together, the *luc* expressing strains AH4775 (*luc*) and AH4826 (*luc* + *lux*) in the presence of increasing concentrations of D-Luciferin had greater penetration of the BLI signals through tissue than any of the *lux* only expressing *S. aureus* strains. Moreover, strain AH4826 (*luc* + *lux*) had BLI signals that could be detected through greater than 2 cm of tissue.

### *In vivo* BLI signals of *lux* versus *luc* in a mouse model of *S. aureus* bacteremia

**Figure 4:**
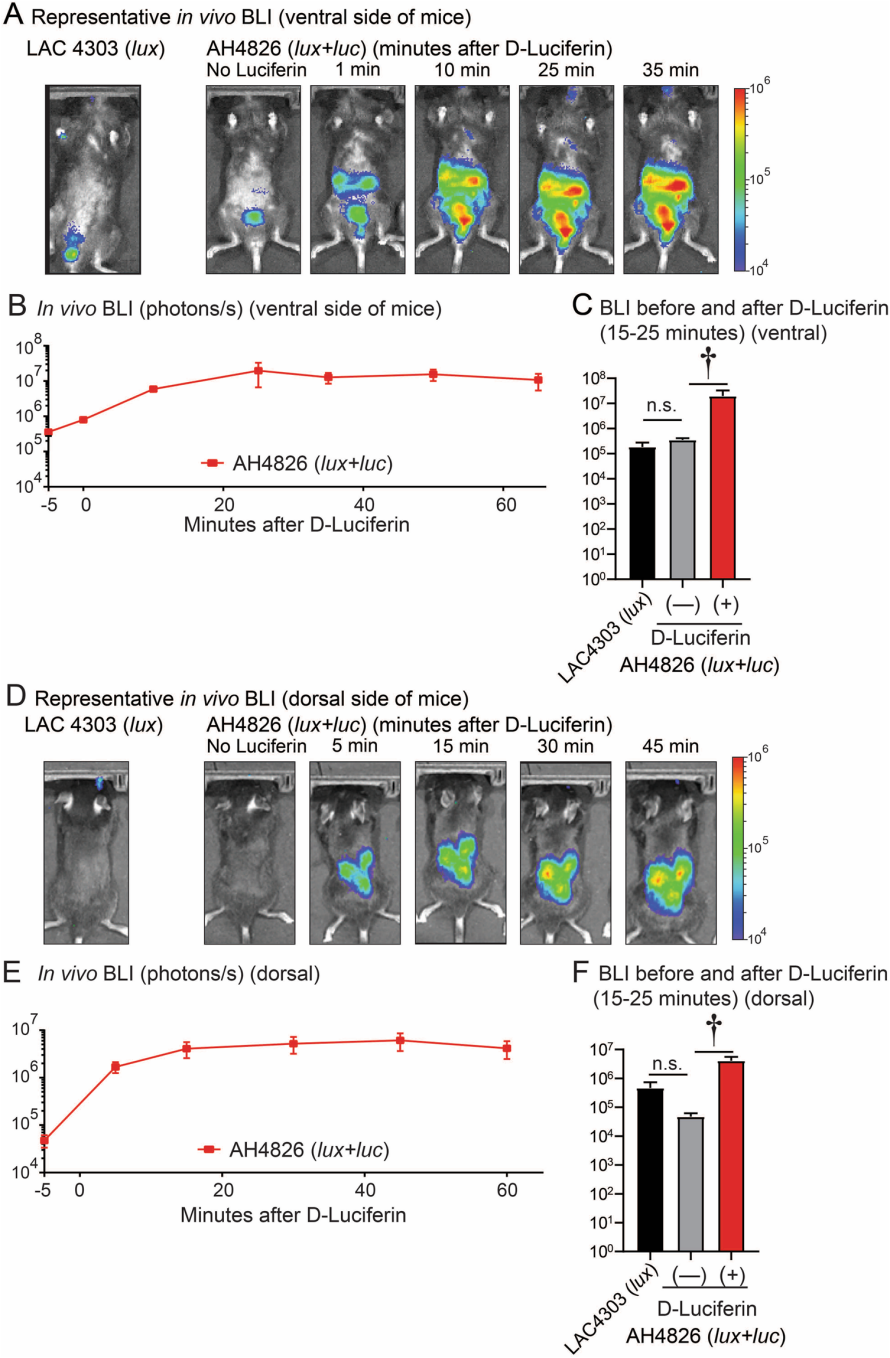
*In vivo* BLI of *lux* versus *luc* in a *S. aureus* bacteremia mouse model. A high inoculum (1 × 10^7^ CFU, 20% lethal dose) of AH4826 (*lux* + *luc*) or LAC4303 (*lux*) was injected intravenously in mice (n = 3 mice/group). On day 3, *in vivo* BLI signals were acquired before (−5 minutes) and at the indicated time points (up to 65 minutes) after administration of D-Luciferin (150 mg/kg s.c.) (IVIS Lumina III, PerkinElmer). (**A,D**) Representative images of *in vivo* BLI of the ventral (**A**) and dorsal (**D**) sides of the mice. (**B,E**) *In vivo* BLI signals (photons/s) ± SEM of the ventral (**B**) and dorsal (**E**) sides of mice inoculated with AH4826 ± administration D-Luciferin (150 mg/kg s.c.). (**C,F**) *In vivo* BLI signals (photons/s) ± SEM of the ventral (**C**) and dorsal (**F**) sides of mice inoculated with AH4826 (*lux* + *luc*) or LAC4303 (*lux*) ± administration of D-Luciferin (150 mg/kg s.c.) at 15–25 minutes prior to imaging the mice. ^†^*P* < 0.01 between indicated groups, as calculated by a 2-tailed Mann–Whitney *U* test (**C,F**).

To evaluate whether the improved tissue penetration using *luc*, compared with *lux*, *in vitro* (Fig. 3) occurred similarly *in vivo*, AH4826 (*lux* + *luc*) and LAC4303 (*lux*) were evaluated in a mouse model of *S. aureus* bacteremia (Fig. 4A–F). Both bacterial strains were inoculated intravenously at a 20–30% lethal dose (1 × 10^7^ CFU) (Fig. S3). On day 3 post-inoculation, the *lux* only representative *in vivo* BLI signals (*i.e*., no D-Luciferin administration) of AH4826 (*lux* + *luc*) and LAC4303 (*lux*) were relatively dim and could be only be detected from the bladder on the ventral sides of the mice (Fig. 4A) and from the kidneys on the dorsal side of the mice (Fig. 4D). To evaluate the *luc in vivo* BLI signals, on day 3 post-inoculation of AH4826 (*lux* + *luc*), D-Luciferin was administered subcutaneously and the mice were imaged for various time points up to 65 minutes. The administration of D-luciferin resulted in detectable *in vivo* BLI signals from the bladder as well as other internal organs in the abdominal cavity such as the kidneys and liver (no signals were detected from the chest) (Fig. 4A,D). Regarding the timeframe for optimal detection of *in vivo* BLI signals after D-Luciferin administration, the *in vivo* BLI signals increased over the first 10 minutes, peaking around 15 minutes and then remained at a similar level for up to 65 minutes, when imaging the mice was arbitrarily discontinued (Fig. 4B,E). Without the addition of D-Luciferin, there was no statistical differences in the *lux in vivo* BLI signals from AH4826 (*lux* + *luc*) and LAC4303 (*lux*) on the ventral or dorsal sides of the mice (Fig. 4C,F). The *in vivo* BLI signals from the abdominal cavity were significantly and markedly higher (155-fold on ventral side and 114-fold on dorsal side of the mice) with AH4826 (*lux* + *luc*) after D-luciferin administration compared with the *lux* only signals of AH4826 (*lux* + *luc*) (*P* < 0.01) (Fig. 4C,F). Taken together, administration of D-Luciferin to AH4826 (*lux* + *luc*)-infected mice resulted in a marked increase of the *in vivo* BLI signals from internal organs compared with the *lux* only BLI signals of AH4826 (*lux* + *luc*) or LAC4303 (*lux*). In addition, the optimal timeframe for *in vivo* BLI of the mice after D-Luciferin administration was ~15–25 minutes and this timeframe was used in subsequent experiments.

### *In vivo* BLI signals of *lux* versus *luc* in other animal models of *S. aureus* infection

**Figure 5:**
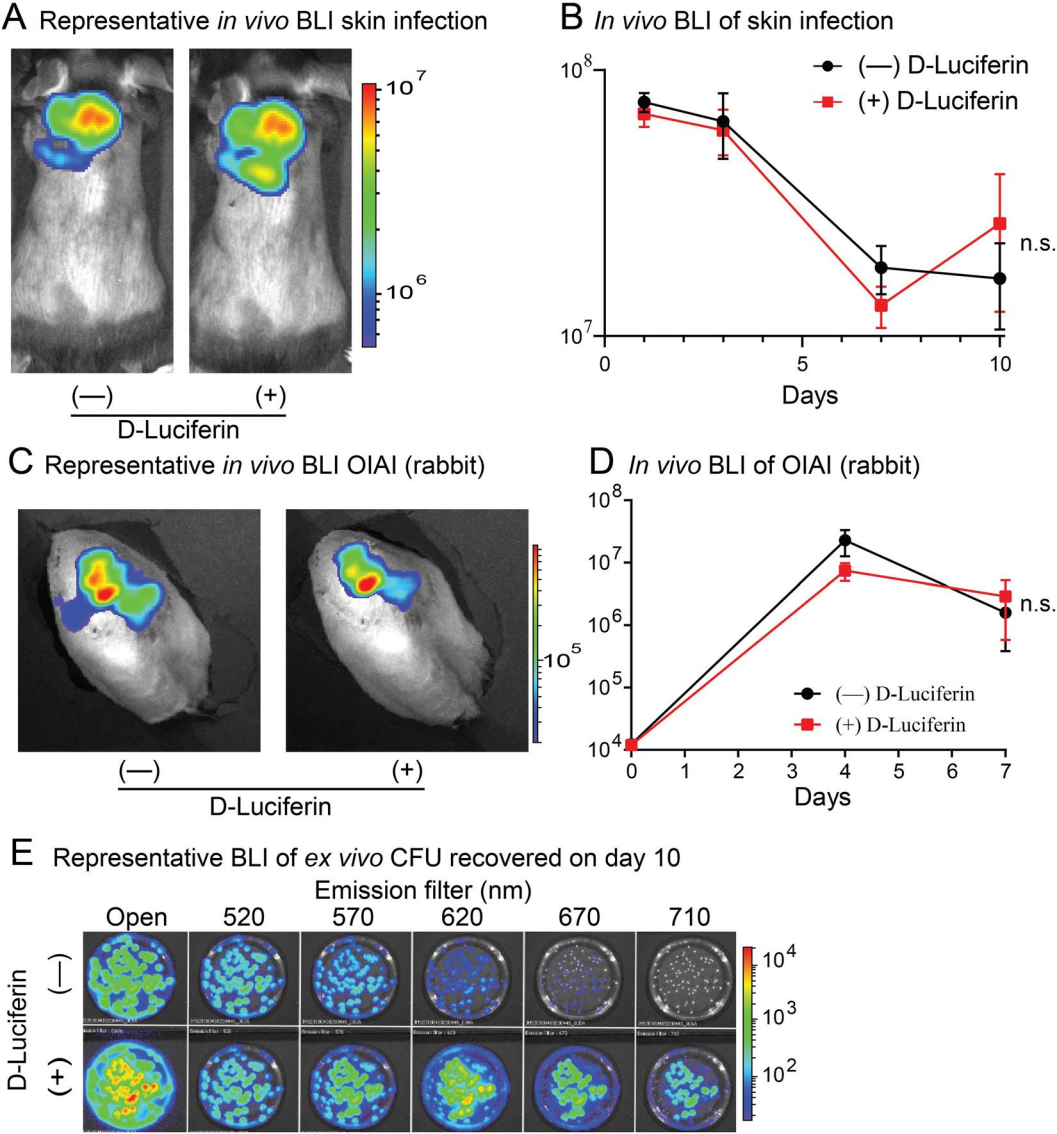
*In vivo* BLI of *lux* versus *luc* in additional models of *S. aureus* infection. (**A,B)** A mouse model of *S. aureus* skin infection was performed with inoculation of AH4826 (*lux* + *luc*) **(**1 × 10^8^ CFU) i.d. into the back skin of mice (n = 10 mice/group). *In vivo* BLI imaging was performed ± administration of D-Luciferin (150 mg/kg s.c.) at 15–25 minutes prior to imaging the mice (n = 10/group). **(A)** Representative *in vivo* BLI. (**B**) *In vivo* BLI signals (photons/s) ± SEM. (**C–E**) A rabbit model of *S. aureus* orthopaedic implant associated infection (OIAI) was performed with inoculation of AH4826 (*lux* + *luc*) **(**1 × 10^4^ CFU) into a femoral intramedullary canal prior to surgical placement of an orthopaedic-grade titanium locking peg (n = 6 rabbits/group) and *in vivo* BLI imaging was performed ± administration of D-Luciferin (150 mg/kg s.c.) at 15–25 minutes prior to imaging the rabbits. **(C)** Representative *in vivo* BLI. **(D**) *In vivo* BLI signals (photons/s) ± SEM. (**E**) Skin tissue from day 10 was homogenized and *ex vivo* CFU of AH4826 (*lux* + *luc*) was cultured on bacterial culture petri dishes overnight (n = 5 replicates) and BLI was performed for a 30 second acquisition time at 37 °C ± the addition of D-Luciferin (600 ng/200 µL PBS pipetted directly onto the surface of the plates) on an IVIS Lumina III (PerkinElmer) and representative BLI signals are provided with no filter (open) and with 520, 570, 620, 670 and 710 nm emission filters. *P < 0.05 between ± administration of D-Luciferin, as calculated by a 2-way ANOVA (**B,D**). n.s. = not significant.

Since AH4826 (*lux* + *luc*) after D-Luciferin administration resulted in enhanced *in vivo* BLI signals in the mouse model of *S. aureus* bacteremia *in vivo* (Fig. 4), similar enhancement might occur in other preclinical models of *S. aureus* infection. A previously described *S. aureus* skin infection model was employed in which a bioluminescent *S. aureus* strain was inoculated intradermally (i.d.) into the dorsal back skin of mice and the ensuing *in vivo* BLI signals were measured (IVIS Lumina III)^22^. This model was performed with AH4826 (*lux* + *luc*) (1 × 10^8^ CFU) ± D-Luciferin administration (Fig. 5A,B). There were no differences between the *in vivo* BLI signals ± D-Luciferin for the entire 10-day course of infection.

As an alternative preclinical *S. aureus* infection model, a *S. aureus* orthopaedic implant associated infection (OIAI) model in rabbits was also evaluated^59^. This model involved performing a medial parapatellar arthrotomy on the right rabbit knee, drilling a hole in the distal femur, inoculating a bioluminescent *S. aureus* strain into the intramedullary femoral canal and surgically placing of an orthopaedic-grade titanium locking peg into the canal prior to closure. The ensuing *in vivo* BLI signals from the infected post-surgical knees were measured noninvasively (IVIS Lumina III). This model was performed with AH4826 (*lux* + *luc*) (1 × 10^4^ CFU) ± D-Luciferin administered prior to *in vivo* BLI (Fig. 5C,D). There was no difference between the *in vivo* BLI signals ± D-Luciferin for the entire 7-day course of the *S. aureus* OIAI.

To determine whether the AH4826 (*lux* + *luc*) bacteria maintained the *luc* plasmid construct after the *in vivo* infection, the infected skin tissue on day 10 (Fig. 5B) was homogenized and *ex vivo* CFU were cultured on plates overnight. BLI of the plates was performed ± the addition of D-Luciferin to the surface of the plates with no filter (open) and with 520, 570, 620, 670 and 710 nm emission filters (IVIS Lumina III) as in Fig. 3A (Fig. 5E). The same pattern of BLI signals with and without the addition of D-Luciferin was observed as was with the *in vitro* mid-logarithmic phase bacteria in Fig. 3A, indicating that the *ex vivo* bacteria maintained the *luc* construct following the 10-day *in vivo* skin infection. Taken together, in both the *S. aureus* skin infection mouse model and the *S. aureus* OIAI rabbit model, the administration of D-Luciferin did not further enhance the *in vivo* BLI signals of AH4826 (*lux* + *luc*). Furthermore, the lack of enhancement of the BLI signals by administration of D-Luciferin was not due to loss of the *luc* plasmid construct *in vivo* as *ex vivo* bacterial cultures had the same pattern of BLI signals ± D-Luciferin as the initial mid-logarithmic phase bacteria prepared *in vitro*.

### Anatomical co-registration of *in vivo* BLI signals of *lux* versus *luc*

**Figure 6:**
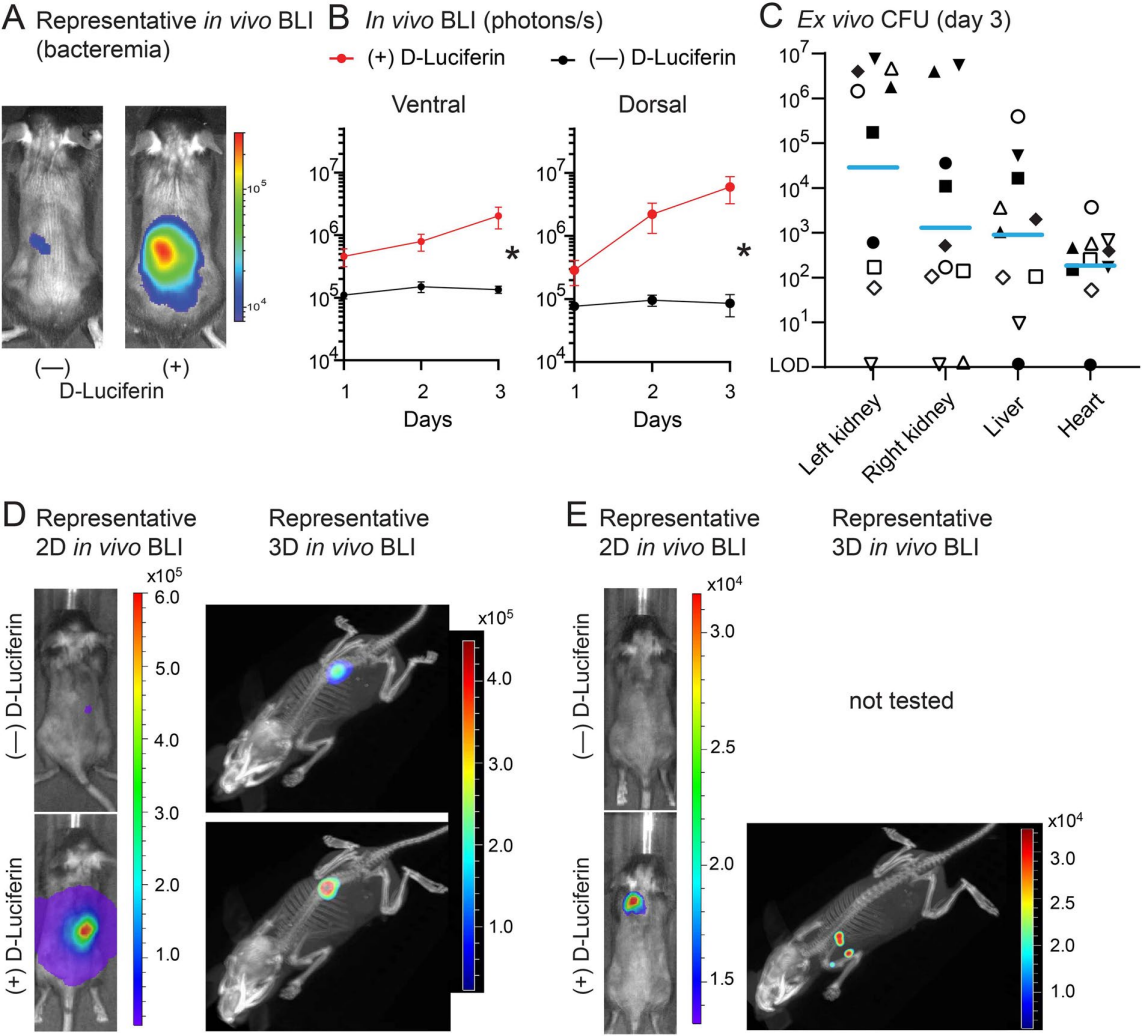
3D localization *in vivo* BLI of *lux* versus *luc* in a *S. aureus* bacteremia mouse model. A sub-lethal inoculum (1 × 10^6^ CFU) of AH4826 (*lux* + *luc*) was injected intravenously in mice (n = 10 mice/group) and mice were imaged ± administration of D-Luciferin (150 mg/kg s.c.) at 15–25 minutes prior to *in vivo* BLI. (**A**) Representative *in vivo* BLI images of the dorsal sides of the mice on day 3 obtained using the 2D IVIS Lumina III imaging system (PerkinElmer). (**B**) *In vivo* BLI signals (photons/s) ± SEM of the ventral (left) and dorsal (right) sides of mice. (**C**) Mice were euthanized on day 3 and right and left kidneys, liver and heart were harvested and homogenized and the *ex vivo* CFU were enumerated after overnight culture on plates. Each symbol is an individual mouse. Light blue bars = geometric mean. LOD = limit of detection [10 CFU]). (**D,E**) Representative 2D and 3D *in vivo* BLI images of the dorsal sides of the mice (n = 10 mice) on day 3 obtained using the IVIS Spectrum-CT imaging system (PerkinElmer). *P < 0.05 between ± administration of D-Luciferin, as calculated by a 2-way ANOVA (**B**).

Since *in vivo* BLI of AH4826 (*lux* + *luc*) after D-Luciferin administration resulted in enhanced signal detection in the mouse model of *S. aureus* bacteremia *in vivo* from bacteria that disseminated to internal organs (Fig. 4), this model was used to determine whether 3D *lux* and *luc* signals could be co-registered with computed tomography (CT) images of the mice using the IVIS Spectrum-CT imaging system (PerkinElmer). The *S. aureus* bacteremia model with AH4826 (*lux* + *luc*) was performed as in Fig. 4 but with a lower (sub-lethal) inoculum (1 × 10^6^ CFU) because the 3D IVIS Spectrum-CT imaging system has greater sensitivity for detecting *in vivo* BLI signals compared with the 2D IVIS Lumina III imaging system, which was used to acquire all other BLI data. On day 3 post-inoculation, the *in vivo* BLI signals of *lux* only (*i.e*., no D-Luciferin administration) resulted in dim bladder signals on the ventral sides of the mice and dim signals from the kidneys on the dorsal sides of the mice (Fig. 6A). Additional representative images of 3 of the 10 mice in this experiment (with similar results) imaged on the 2D IVIS Lumina III are also provided (Fig. S4). In contrast, after D-Luciferin administration, substantially increased *in vivo* BLI signals could be detected from the middle and lower back on the dorsal sides of the mice (Fig. 6A). Indeed, the *in vivo* BLI signals after D-luciferin were 4-fold (day 1) to 15-fold (day 3) greater than without D-Luciferin on the ventral sides of the mice and 4-fold (day 1) to 70-fold (day 3) greater than without D-Luciferin of the dorsal sides of the mice (Fig. 6B). On day 3, mice were euthanized and *ex vivo* CFU were enumerated after overnight culture of organ homogenates of the right kidney, left kidney, liver and heart. Overall, there were higher CFU isolated from the left and right kidneys (geometric mean = 2.89 × 10^4^ CFU and 1.29 × 10^3^ CFU, respectively) compared with CFU isolated from the liver (geometric mean = 8.97 × 10^2^ CFU) or heart (geometric mean = 1.86 × 10^2^ CFU) (Fig. 6C).

To provide noninvasive information about the anatomic source of the *in vivo* BLI signals, *in vivo* BLI and CT images of 5 mice were co-registered on day 3 after the bacterial inoculation (Fig. 6D,E). Representative 2D *in vivo* BLI images (Fig. 6D [left panels]) and 3D *in vivo* BLI signals co-registered with the CT image (Fig. 6D [right panels] and Movies S1 and S2) are shown of a single mouse that had a dim signal from the right kidney without D-Luciferin (Fig. S5A and Movie S1) and a markedly increased signal from the right kidney after D-Luciferin administration (Fig. S5B and Movie S2). In addition, representative 2D *in vivo* BLI images (Fig. 6E [left panels]) and 3D *in vivo* BLI signals and CT image co-registration (Fig. 6E [right panels]) of a single mouse that had no signals without D-Luciferin and had a detectable signal from the right axillary/chest regions after D-Luciferin administration (Fig. S6 and Movie S3). Taken together, 3D *in vivo* BLI along with CT image co-registration was able to localize the source of the *lux* and *luc* signals. Importantly, administration of D-Luciferin to provide additional *luc* signals provided the new capability to visualize and co-register the source of *in vivo* BLI signals that could not be detected at all with the *lux* signals alone.

### Sensitivity and duration of the *in vivo* BLI signals of the *luc* construct in the mouse model of *S. aureus* bacteremia

**Figure 7:**
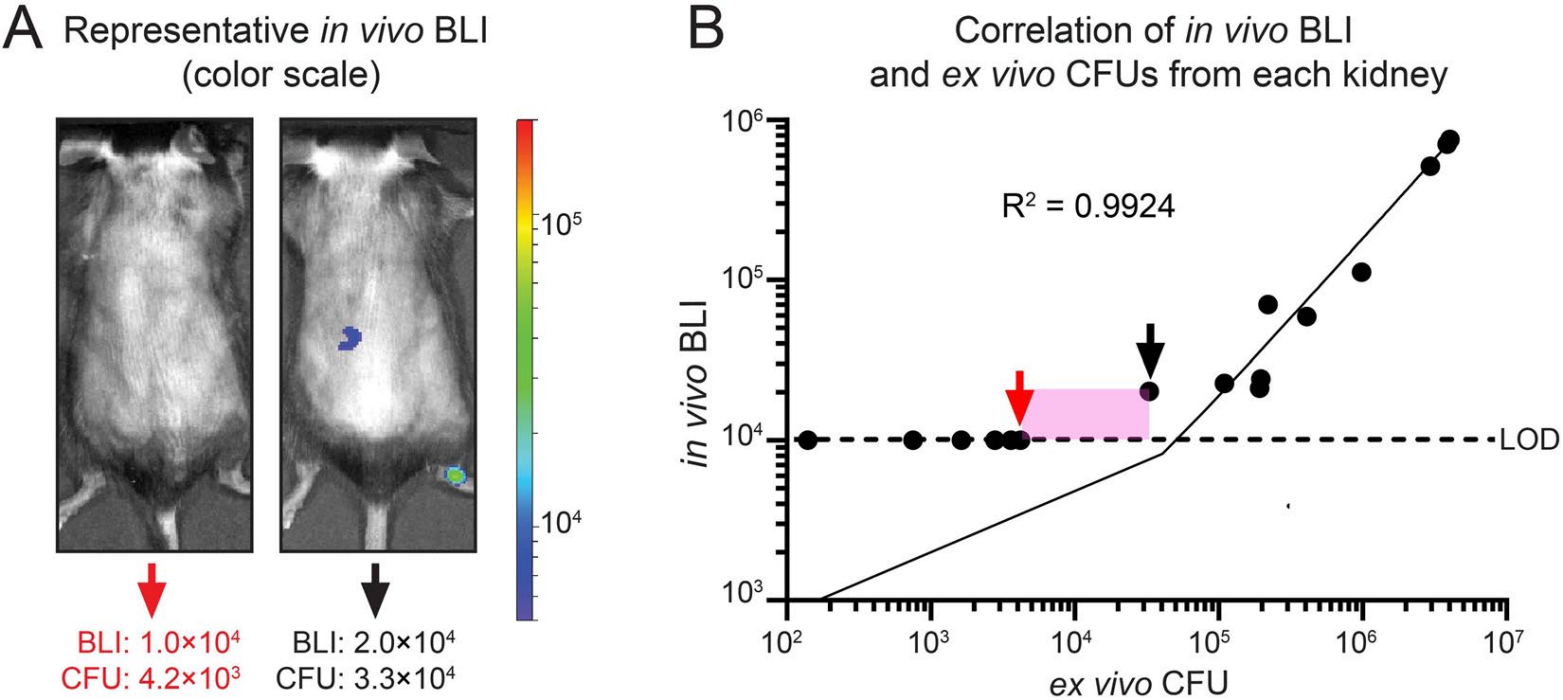
The sensitivity of detection of AH4775 (*luc*) by *in vivo* BLI in the *S. aureus* bacteremia mouse model. Sixteen hours post-intravenous inoculation of 1 × 10^6^ CFU of AH4775 (*luc*) in mice (n = 8 mice), D-Luciferin (150 mg/kg s.c.) was administered and after 20 minutes *in vivo* BLI signals was performed on the dorsal sides of the mice and *in vivo* BLI signals were acquired as total flux (photons/s) within region of interests corresponding to the right and left kidneys (IVIS Lumina III). Mice were then immediately euthanized and the right and left kidneys were separately isolated to determine the *ex vivo* CFU. (**A**) Representative images of *in vivo* BLI signals of a mouse with the highest CFU but below the level of detection (LOD) of the IVIS Lumina III (left, red arrow) and a mouse with the lowest detectable *in vivo* BLI signal from the left kidney that was above the LOD (right, black arrow). (**B**) Correlation between *in vivo* BLI (total flux [photons/s]) and *ex vivo* CFU from the right and left kidneys of the mice (n = 8 mice) with the red and black arrows denoting the representative mice shown in (**A**), respectively. LOD = 1 × 10^4^ photons/s (horizontal black dashed line). The pink boxed area represents the range of sensitivity of detection from the highest CFU that was not detected (4.2 × 10^3^ CFU) to the lowest CFU that was detected (3.3 × 10^4^ CFU) by *in vivo* BLI. The linear regression line and correlation coefficient of determination (R^2^ = 0.9924) of *in vivo* BLI and *ex vivo* CFU of AH4775 (*luc*) are shown.

Since the *in vivo* BLI signals from strain AH4826 (*lux* + *luc*) after D-Luciferin administration were more than 100-fold greater than *S. aureus* strains expressing only the *lux* construct (Fig. 4C,F), the lower limit of sensitivity for *in vivo* BLI signals of AH4775 (*luc*) was determined. To accomplish this, the mouse model of *S. aureus* bacteremia was performed with the sub-lethal inoculum (1 × 10^6^ CFU) of AH4775 (*luc*). At 16-hours post-intravenous inoculation, D-Luciferin was administered and after 20 minutes, *in vivo* BLI signals from regions of interests overlying the right and left kidneys on the dorsal sides of the mice were acquired (IVIS Lumina III) with a longer (5 minute) acquisition time. All mice were immediately euthanized, each kidney was harvested and *ex vivo* CFU were enumerated. The *in vivo* BLI and *ex vivo* CFU from each of the right and left kidneys were graphed on a dot plot (Fig. 7A,B). Using the IVIS Lumina III imaging system with the lower limit of detection (LOD) of 1 × 10^4^ photons/s, there was a range of sensitivity in which the *in vivo* BLI signals reached the LOD for the mouse with 4.2 × 10^3^ CFU isolated from the kidneys (as well as all mice with lower *ex vivo* CFU) whereas the *in vivo* BLI signals of the mouse with 3.3 × 10^4^ CFU isolated from the kidneys could be detected (as well as all mice with higher *ex vivo* CFU) (Fig. 7A,B). Therefore, the LOD for *in vivo* BLI signals of AH4775 (*luc*) was approximately 3 × 10^4^ CFU. Importantly, there was a high level of correlation between *in vivo* BLI and *ex vivo* CFU of AH4775 (*luc*) (correlation coefficient of determination of R^2^ = 0.9924) (Fig. 7B). In addition, it is important to assess how long can the bioluminescent signals of AH4775 (*luc*) be detected *in vivo*. Therefore, as a proof-of-concept, the same mouse model of *S. aureus* bacteremia was performed in 2 representative mice with the sub-lethal inoculum (1 × 10^6^ CFU) of AH4775 (*luc*) and the *in vivo* BLI signals from the dorsal sides of the mice were monitored weekly. *In vivo* BLI imaging was performed 20 minutes after D-Luciferin administration from the dorsal backs of the mice on a weekly basis and the experiment was arbitrarily ended at 3 weeks because bioluminescent signals were still detected in both mice (Fig. S7A). At the 3-week time point, the mice were euthanized and *ex vivo* CFU from the kidneys were isolated and cultured on plates. The *in vivo* BLI and *ex vivo* CFU from each of the right and left kidneys were graphed on a dot plot (Fig. S7B). The plates were then sprayed with D-Luciferin and imaged in the IVIS Lumina III and in one mouse 100% of the *ex vivo* CFU of AH4775 (*luc*) on the plates from both kidneys still emitted a bioluminescent signal whereas the other mouse had 96% and 64% of *ex vivo* CFU of AH4775 (*luc*) on the plates from the left and right kidneys, respectively, still emitted a bioluminescent signal (Fig. S7C). These data indicate that the *in vivo* BLI signals from AH4775 (*luc*) could still be detected for at least 3 weeks of an *in vivo* bacteremia infection and the *luc* plasmid construct was relatively stable, as 64–100% of the *ex vivo* bacteria isolated still maintained the ability to produce bioluminescent signals.

### Mouse model of a mixed *S. aureus* and *P. aeruginosa* wound infection mouse model

**Figure 8:**
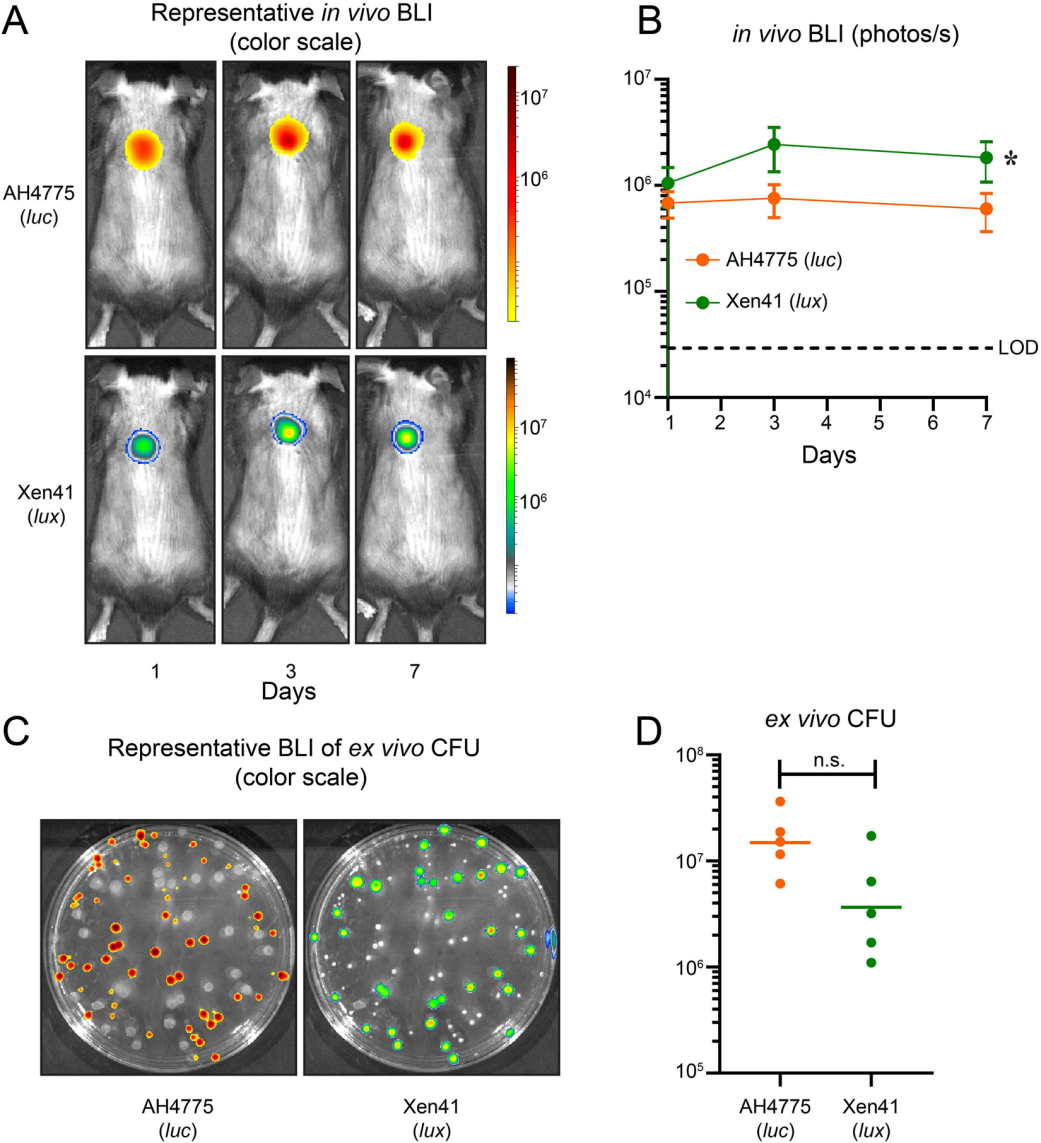
Dual *in vivo* BLI monitoring of a mixed *S. aureus* and *P. aeruginosa in vivo* wound infection mouse model. A excisional wound mixed infection model by performing a 6-mm punch biopsy on the backs of mice and the wound beds were inoculated with *S. aureus* AH4775 (*luc*) (2 × 10^6^ CFU) and *P. aeruginosa* Xen41 (*lux*) (2 × 10^5^ CFU) (n = 5 mice). The mixed wound infection was followed for 7 days and CFU from the wounds were harvested, plated, cultured and enumerated. To distinguish between the *in vivo* BLI signals of AH4775 (*luc*) versus Xen41 (*lux*), the respective 670 nm and 520 nm emission filters (IVIS Lumina III) were used because there was no overlap between the *luc* and *lux* bioluminescent signals. (**A**) Representative images of the *in vivo* BLI signals from AH4775 (*luc*) and Xen41 (*lux*) from the wounds. (**B**) Mean *in vivo* BLI signals of AH4775 (*luc*) and Xen41 (*lux*) quantified as total flux (photons/s) ± SEM. (**C**) Representative *in vivo* BLI of bacterial culture plates possessing CFU of AH4775 (*luc*) and Xen41 (*lux*) using the 670 nm and 520 nm emission filters, respectively. (**D**) Mean CFU of AH4775 (*luc*) and Xen41 (*lux*), with horizontal bars = geometric mean. *P < 0.05 between AH4775 (*luc*) and Xen41 (*lux*), as calculated by a 2-way ANOVA (**C**). n.s. = not significant (*P* = 0.095), as calculated by a 2-tailed Mann–Whitney *U* test (**D**).

The AH4775 (*luc*) strain might also represent a technological advance to study the *in vivo* dynamics of the bacterial burden in a mixed infection model with different *lux*-expressing bacterial species, if the wavelengths of *in vivo* BLI signals from *luc* and *lux* signals could be spectrally unmixed. As a proof-of-concept, a mouse model of a mixed full-thickness wound infection with *S. aureus* and *P. aeruginosa* was modified from a previously established model in which the *S. aureus* inoculum was 10-fold higher than the *P. aeruginosa* inoculum^60,61^. For this experiment, a 6-mm punch biopsy excisional wound was performed on the dorsal backs of mice and the wound bed was immediately inoculated with *S. aureus* AH4775 (*luc*) (2 × 10^6^ CFU) and *P. aeruginosa* Xen41 (*lux*) (2 × 10^5^ CFU) (PerkinElmer). After D-Luciferin (150 mg/kg s.c.) administration and performing *in vivo* BLI of the mice, it was determined that the 670 nm and 520 nm emission filters of the IVIS Lumina III were able to spectrally unmix the *luc* and *lux* signals, respectively, with no appreciable overlap of the bioluminescent signals. Therefore, *in vivo* BLI signals of AH4775 (*luc*) and Xen41 (*lux*) were monitored using these two filters over the course of the 7-day infection (Fig. 8A,B). The *in vivo* BLI signals of AH4775 (*luc*) remained very stable with a slight decrease (12%) in signals from 6.82 × 10^5^ ± 1.9 × 10^5^ photons/s on day 1 to 6.02 × 10^5^ ± 2.3 × 10^5^ photons/s on day 7. In contrast, the *in vivo* BLI signals of Xen41 (*lux*) increased by approximately 2-fold from 1.0 × 10^6^ ± 0.42 × 10^6^ photons/s on day 1 to 2.44 × 10^6^ ± 1.1 × 10^6^ photons/s on day 3 and 1.83 × 10^6^ ± 0.75 × 10^6^ on day 7. It should be noted that the *in vivo* BLI signal intensity for AH4775 (*luc*) in the model was lower than Xen41 (*lux*), which was likely due to the use of the 670 nm filter (which was above the peak wavelength of the *luc* signal following D-Luciferin administration [Table 2]) or due to less bioavailability of D-Luciferin at the site of the mixed infection in this *in vivo* wound model. On day 7, the mice were euthanized and *ex vivo* CFU were isolated and cultured on plates. The plates were then sprayed with D-Luciferin and imaged (IVIS Lumina III) using the same 670 nm and 520 nm emission filters to distinguish between CFU of AH4775 (*luc*) and Xen41 (*lux*), respectively (Fig. 8C). The CFU were enumerated and there was a non-significant trend toward slightly increased CFU of AH4775 (*luc*) (geometric mean: 1.5 × 10^7^ CFU) compared with Xen41 (*lux*) (geometric mean: 3.2 × 10^6^ CFU) (*P* = 0.095) (Fig. 8D). Taken together, these data indicate that a *luc*-expressing *S. aureus* strain could be used with a different *lux*-expressing bacterial strain (*e.g*., *P. aeruginosa* Xen41) to noninvasively and longitudinally monitor the dynamics of the bacterial burden of each bacterial strain in a mixed infection model.

## Discussion

*S. aureus* bacteremia infections are a major clinical problem, especially since the mortality has remained extremely high and the treatment has been complicated by virulent and multi-drug resistant MRSA strains^3–6^. To better study these infections in preclinical animal models, we generated new *S. aureus* strains in the well-studied community-acquired MRSA strain USA300 LAC background that express *lux*, CBR-*luc* or both *lux and* CBR-*luc* in the same strain, including AH4807 (*lux*), AH4775 (*luc*) and AH4826 (*lux* + *luc*), respectively. In AH4775 (*luc*) and AH4826 (*lux* + *luc*), the CBR-*luc* plasmid construct (with the *luc* gene is expressed under the strong constitutive *S. aureus* promoter *hprK/lgt*) was relatively stable, as it was maintained in the majority of progeny after a 3-week *in vivo* experiment. To the best of our knowledge, this is the first report that has successfully been able to generate a CBR-*luc* expressing *S. aureus* strain AH4775 (*luc*) and a dual *lux* and CBR-*luc* expressing *S. aureus* strain AH4826 (*lux* + *luc*).

We found that AH4807 (*lux*) with the newly generated *luxCDABEG* construct integrated in the *S. aureus* chromosome had relatively similar *in vitro* bacterial growth and bioluminescent signals as previously generated *lux* expressing *S. aureus* strains USA300 LAC::*lux*, LAC4303 (*lux*) and Xen36 (*lux*) (Figs 2 and S1). The *lux* and *luc* constructs could also be introduced into other *S. aureus* strains, including USA400 MW2 and Newman, demonstrating the broader utility of these constructs. While LAC4303 (*lux*) yielded the highest luminescence over time, the new AH4807 (*lux*) construct was more stably integrated in the chromosome (Fig. S1). Regarding tissue penetration, LAC4303 (*lux*) and Xen36 (*lux*) could be visualized through approximately 5 mm of tissue (sliced ham) whereas none of the signals from the *lux* expressing strains could be visualized through 10.5 mm of tissue. However, after adding D-Luciferin to cultures of AH4775 (*luc*) and AH4826 (*lux* + *luc*), there was markedly enhanced bioluminescent signals in the 96-well plate assay (Fig. 2) and greatly increased tissue penetration, allowing visualization through >2 cm of tissue with AH4826 (*lux* + *luc*). Therefore, the addition of the *luc* construct provided the improved capability of detecting bioluminescent signals through an increased thickness of tissue to a much greater extent than any of the *S. aureus lux* expressing strains.

Given the increased bioluminescent signals and tissue penetration of AH4826 (*lux* + *luc*) *in vitro*, we investigated this particular strain in an *in vivo* model of *S. aureus* bacteremia. Without the administration of D-Luciferin, only dim *in vivo* BLI signals from *lux* could be detected from the bladder and kidneys on the ventral and dorsal sides of the mice, respectively. However, following administration of D-Luciferin, substantially increased *in vivo* BLI signals (up to 2 logs) could be visualized from the kidneys, liver and bladder on the ventral sides of the mice as well as increased signals from the kidneys on the dorsal sides of the mice. Most impressively, the 3D *in vivo* BLI co-registration with the CT imaging resulted in improved detection of *in vivo* BLI signals from the kidneys. Moreover, in a single mouse there was a focus of a bacterial dissemination in the right axillary/chest region that could only be detected after administration of D-Luciferin. Taken together, the administration of D-Luciferin and ensuing *luc* signals greatly enhanced the capability of detecting *in vivo* BLI signals from bacteria that disseminated to the organs and tissues in the mouse model of *S. aureus* bacteremia. In the bacteremia model, the *luc*-expressing *S. aureus* strain AH4775 (*luc)*, following D-Luciferin administration, had a high level sensitivity of detection of the bacteria burden in the kidneys by *in vivo* BLI imaging, allowing as few as 3.3 × 10^4^ CFU to be detected whereas 4,200 CFU could no longer be detected in the IVIS Lumina III. Furthermore, the *in vivo* BLI signals of AH4775 (*luc)* after D-Luciferin administration could be detected in the kidneys at least 3-weeks after the bacterial inoculation and most of *ex vivo* CFU maintained the ability to produce *luc* bioluminescent signals after the 3-week *in vivo* experiment (ranging from 64–100%). Nonetheless, for long-term *in vivo* studies, the *in vivo* BLI signals of the plasmid-based *luc* construct might underestimate the actual *in vivo* bacterial burden and therefore our future studies will evaluate whether the *luc* construct could be inserted into the bacterial chromosome for improved stability.

The improved ability to detect *in vivo* BLI signals in the mouse model of *S. aureus* bacteremia model is likely due to a combination of increased light production by the *luc* construct as well as the longer wavelength of light produced (614 nm) that enhanced tissue penetration. In the future, alternative substrates for the same *luc* construct in AH4775 (*luc*) and AH4826 (*lux* + *luc*) could be evaluated, such as the alkylaminoluciferins, aminoluciferins, naphthyl-based and AkaLumine-HCl luciferin analogs, luciferase substrates that can produce even longer wavelengths of light (up to and exceeding 730 nm), possibly allowing even greater tissue penetration than with D-Luciferin^58,62–64^. These other substrates might be particularly useful in mouse models of *S. aureus* bacteremia to detect the low bacterial CFU that are present in liver and heart (~10^2^ CFU range) and potentially other disseminated organs and tissues. These analogs (administered systemically or perhaps locally [*e.g*., topical application or by local injection at the site of infection]) might also be able to enhance the signal of *luc*-expressing *S. aureus* strains over that of *lux*-expressing *S. aureus* strains, which might be especially useful in enhancing the signals in the mouse model of *S. aureus* skin infection and the rabbit orthopaedic implant model. These further enhancements and optimization of the *luc* signals will be the subject of our future work. In addition, the specific *S. aureus luc* construct developed in this study could be further modified with species-specific promoters and stable plasmid constructs so that it could be used in other Gram-positive bacteria, such as *S. epidermidis* (in which existing *lux* signals are dim, especially in biofilms^38,46^), *Streptococcus pyogenes* and *Streptococcus pneumoniae* to provide an alternative to *lux* expressing strains for better *in vivo* BLI detection in preclinical animal models of infection with these bacteria.

For *in vivo* BLI, the *S. aureus luc* signals were expected to be superior to *lux* signals because of the longer peak wavelength of the bioluminescent signals from CBR-*luc* that would have better tissue penetration. Indeed, CBR-*luc* has been shown to enhance *in vivo* BLI signals in preclinical mouse models with other microorganisms, including *Listeria monocytogenes*, *Mycobacterium tuberculosis*, and *Candida albicans*^47,50,51^. However, when AH4826 (*lux* + *luc*) was evaluated in a *S. aureus* skin infection mouse model and in a *S. aureus* OIAI rabbit model, the administration of D-Luciferin did not enhance the *in vivo* BLI signals produced by *lux* alone. Although the reason for this is not entirely clear, the bioluminescent signals from *luc* are dependent on the bioavailability of the D-Luciferin substrate, which was likely higher in the organs in the bacteremia model compared with the sites of the bacterial infection in the skin or OIAI models. This and other possibilities will be evaluated in our future work as they are beyond the scope of this initial report. Nonetheless, our results strongly suggest that a bioluminescent *S. aureus* strain such as AH4826 (*lux* + *luc*) would be extremely versatile as it would take advantage of signals from both *lux* and *luc* for improved *in vivo* BLI in different models of *S. aureus* infection.

It should also be mentioned that *lux* expressing bacteria and *luc* expressing bacteria can be used in conjunction with *in vivo* BLI as the different wavelengths of bioluminescent light from each strain can be spectrally unmixed. For *S. aureus*, the interactions of two or more bacteria by using *luc* versus *lux* expressing bacteria has important translational relevance as many *S. aureus* infections in humans are polymicrobial, including burns, chronic wounds, diabetic foot ulcers and surgical site infections^65^. Specifically, in various preclinical models of these polymicrobial infections, *in vivo* BLI could be used to simultaneous study *S. aureus* strain AH4775 (*luc*) along with another *lux* expressing bacterial strain (*e.g*., *Pseudomonas aeruginosa, Streptococcus pneumoniae* and *Streptococcus pyogenes*, *Haemophilus influenzae* or *Enterococcus faecalis*) to provide new insights into cooperative and competitive interactions between the different bacterial species with respect to bacterial growth, virulence and antibiotic susceptibility. As a proof-of-concept, we performed a mouse model of a mixed full-thickness wound infection with *S. aureus* AH4775 (*luc*) and *P. aeruginosa* Xen41 (*lux*). Using the 670 nm and 520 nm emission filters of the IVIS Lumina III, the *S. aureus luc* signals and *P. aeruginosa lux* signals could be spectrally unmixed, which permitted the noninvasive and longitudinal monitoring of the dynamics of the bacterial burden of each bacterial strain in this mixed infection model. Similar approaches have been previously used to study mutant versus wildtype *M. tuberculosis* strains during a pulmonary infection in mice^47^ and to investigate *Lactobacillus plantarum* versus *Lactococcus lactis* persistence in intestinal compartments of mice^48^.

Taken together, the development of *luc* and *lux* + *luc* expressing *S. aureus* strains provided increased bioluminescent signals with deeper tissue penetration for improved *in vivo* BLI to study organ dissemination during a preclinical *S. aureus* bacteremia infection in mice. This study also provided the proof-of-concept of combining *lux* and *luc* constructs in the same bacterial strain AH4826 (*lux* + *luc*) to optimize the detection of *in vivo* BLI signals in different models of *S. aureus* infections. Therefore, the *luc* and *lux* + *luc* expressing *S. aureus* strains developed in this study represent a technological advance for improved *in vivo* BLI of preclinical *S. aureus* infection models to help provide new insights in the pathogenesis of the infection as well as evaluate novel therapeutic and diagnostic modalities.

## Methods

### Construction of a *lux* expressing *S. aureus* strain

A *luxCDABEG* gene sequence, derived from the bioluminescent bacterial insect pathogen *Photorhabdus luminescens* (GenBank: MYFJ01000025.1), was synthesized (GenScript) with Gram-positive ribosome binding sites at the start of each respective gene, all common restriction enzyme sites were removed and P_CP25_ and P_CAP_ promoters introduced at the start of this operon^66^. This DNA sequence was then amplified using Phusion DNA polymerase (NEB) and primers *lux* promoter (prom) 5′XbaI (GTT GAT TCT AGA GAT CTC GAG ATC TGC AAG ATC C) and *luxG 3*′EcoRI (GAA GTT GAA TTC TTA AAT AAA TTC GAA AGC ATC ACC ATA CAT G). The product was digested with XbaI and EcoRI before ligating into pLL29^52^ to generate pHC125. The ligation reaction was transformed into *E. coli* DH5α, selecting with LB supplemented with 50 μg/mL spectinomycin. Positive clones were identified by bioluminescence, and the plasmids were isolated and electroporated into *S. aureus* RN4220 containing pLL2787^52^. *S. aureus* RN4220 colonies with chromosomally integrated pHC125 were selected on TSB supplemented with 1 μg/mL tetracycline. The integrated *luxCDABEG* cassette was then transduced into *S. aureus* strains USA300 LAC strain (AH1263^53^), USA400 MW2^54^ and Newman^55^ using phage 11^67^, generating strain AH4807 (*lux*), AH4821 (*lux*) and AH5016 (*lux*), respectively (see Table 1).

### Construction of click beetle luciferase (*luc*)-expressing *S. aureus* strains

A codon optimized version of click beetle red luciferase (*luc*) was synthesized (GenScript) and cloned into the *S. aureus* shuttle vector pCM28^53^ under the control of the *S. aureus hprK/lgt* promoter in multiple steps. Beginning with pCM28 expressing *DsRed* (pHC48^68^), the *sarA* promoter was replaced with the *S. aureus hprK/lgt* promoter. The region upstream of *hprK* was amplified from CA-MRSA LAC strain using primers Plgt 5′*XbaI* (GTT GTT TCT AGA GCC AAC TTG CAT TGT TTG TAG AA) and *Plgt* 3′ *KpnI* (GTT GTT GGT ACC CAA TTG TAT TTA TCC CTA CTC TTA CAT CTC). The resulting product was digested with XbaI and KpnI, and ligated into pHC48 digested with the same enzymes, generating pHC52. The *DsRed* gene was then replaced with the *luc* gene. *Luc* was amplified with primers *Luc* + *RBS* (CTT TAT AAG GAG GAA AAA CAT ATG GTA AAG CGT GAG AAA AAT GTC) and *Luc* 3′EcoRI (CAA CGA ATT CCT AGA TTA TTA CTA ACC GCC GG). An optimized ribosome binding site^69^ was then built onto this sequence with a second round of PCR, using primers *Luc* + *RBS* 5′*KpnI* (GTT TGG TAC CTG ATT AAC TTT ATA AGG AGG AAA AAC ATA TGG T) and *luc* 3′*EcoRI*. The product was digested with KpnI and EcoRI, and ligated into pHC52 digested with the same enzymes, generating pHC123. The plasmid was transformed into *E. coli* DH5α, selecting on LB supplemented with 100 μg/mL ampicillin. The *luc* gene was confirmed by sequencing and the plasmid was then moved into *S. aureus* RN4220 by electroporation, selecting on TSB supplemented with 10 μg/mL chloramphenicol. pHC123 was then transduced into *S. aureus* LAC, MW2 and Newman to generate AH4775 (*luc*), AH5557 (*luc*), AH5556 (*luc*) respectively. In addition, the plasmid was transduced into the *lux* positive strains AH4807 (*lux*), AH4821 (*lux*) and AH5016 (*lux*) to generate AH4826 (*lux* + *luc*), AH5559 (*lux* + *luc*) and AH5558 (*lux* + *luc*), respectively.

### Previously generated bioluminescent *S. aureus* strains

The previously generated *lux* expressing *S. aureus* strains were used. USA300 LAC::*lux* with the *lux* construct in the bacterial chromosome that was generated from the parent community-acquired MRSA USA300 LAC strain obtained from a skin infection outbreak in the Los Angeles County Jail and was kindly provided by Tammy Kielian (University of Nebraska)^15^. LAC4303 (*lux*) (also designated SAP430) with the *lux* construct in the bacterial chromosome that was generated from the parent JE2 strain, which is the MRSA USA300 LAC strain cured of its native plasmids^14,56^. Xen36 (*lux*) with the *lux* construct integrated in a stable plasmid that was generated from the parent methicillin-sensitive *S. aureus* bacteremia isolate Wright (ATCC 49525)^11^.

### Bacterial preparation

All *S. aureus* strains were streaked on tryptic soy agar (TSA) plates (tryptic soy broth [TSB] plus 1.5% bacto agar [BD Biosciences, San Jose, CA]) and grown overnight at 37 °C. Single colonies were selected and cultured in TSB at 240 RPM at 37 °C in a shaking incubator overnight followed a 1:50 subculture at 37 °C in a shaking incubator for 2 hours to obtain mid-logarithmic phase bacteria. For strains AH4775 (*luc*) and AH4826 (*lux* + *luc*), to maintain the *luc* plasmid-based construct, all *in vitro* cultures were performed using TSB in the presence of 10 µg/mL of chloramphenicol. Bacteria were pelleted, washed and re-suspended in either TSB for *in vitro* experiments or PBS for *in vivo* experiments. Absorbance (A_600_) was used to estimate CFU for *in vitro* and *in vivo* experiments, which were verified by overnight culture plating on TSA plates.

*P. aeruginosa* Xen41 was streaked on LB plates (Luria-Bertani broth plus 1.5% bacto agar [BD Biosciences, San Jose, CA]). Single colonies were selected and cultured in LB at 240 RPM at 37 °C in a shaking incubator overnight followed a 1:50 subculture at 37 °C in a shaking incubator for 2 hours to obtain mid-logarithmic phase bacteria.

### *In vitro* growth curves and *lux* versus *luc* bioluminescent signals for Figure S1A and Figure S2

All bioluminescent *S. aureus* strains were grown in TSB overnight at 37 °C in a shaking incubator at 240 rpm. Bacterial cells were harvested by centrifugation (5000 rpm for 10 minutes), washed twice in the same volume of phosphate buffered saline (PBS) and resuspended in TSB. Chloramphenicol (10 μg/mL) was added to cultures when needed to maintain plasmid stability. Absorbance at 600 nm (A_600_) was adjusted to 0.05 for all strains. 200 µL of bacterial culture were added to black 96-well plates with clear bottoms (Corning) and plates were incubated at 37 °C with shaking in a humidified microtiter plate shaker (Stuart). A Tecan Infinite M Plex plate reader was used to periodically measure bacterial growth (A_600_) and luminescence intensity (Integration time 1,000 milliseconds). For Fig. S1A, values from quadruplicate wells were averaged and luminescence was expressed as relative luminescence units corrected to normalized bacterial growth (A_600_). The experiment was repeated three times with biological replicates. For Fig. S2, values from triplicate wells without or with addition of different concentrations (0.125–5 mg/ml) of D-Luciferin (XenoLight D-Luciferin - K^+^ Salt Bioluminescent Substrate, PerkinElmer) were averaged and luminescence was expressed as relative luminescence units (RLU). The experiment was repeated three times with biological replicates.

### Chromosomal integration stability of the *lux* bioluminescent construct for Figure S1B,C

LAC4303 (*lux*) and AH4807 (*lux*) were grown in 5 mL of TSB at 37 °C in a shaking incubator at 240 rpm overnight. One mL was harvested by centrifugation (5000 rpm, 10 minutes), washed twice in the same volume of PBS and resuspended in 700 µL TSB. Cells were diluted up to 10^−6^ and 100 µL were subsequently plated on three TSA plates; one plate was incubated at 43 °C, one at 37 °C and one at 30 °C for 18 hours. To determine whether plasmids are stably integrated into the chromosome, five randomly selected colonies from TSA plates incubated at 43 °C, 37 °C and 30 °C were resuspended in PBS, pelleted (5000 rpm, 10 minutes), resuspended in double distilled H_2_O (ddH_2_O) with 0.5 mg/mL lysostaphin (AMBI Products, LLC) and incubated for 45 minutes at 37 °C. 5 µL of the cell lysate was used per PCR reaction (*Taq* DNA Polymerase, NEB). PCR reactions were performed using primers shown in Fig. S1B, and all primer sequences are published^14,52^. Loading dye (TriTrack, Thermo Scientific) was added to all PCR reactions and 6 µL of each PCR reaction were analyzed on a 1% agarose gel, which was stained in an ethidium bromide (Teknova) bath, destained in water and visualized with a UV Transilluminator (G:Box, Syngene) and GeneSys software (Syngene, Version 1.6.6.0). Original agarose gel images were color inverted, cropped and labeled using IrfanView software (Austria, Version 4.53) and Adobe Illustrator (USA, Version 23.0.3).

### *In vitro* growth curves and *lux* versus *luc* bioluminescent signals

AH4807 (*lux*), AH4775 (*luc*), AH4826 (*lux* + *luc*), USA300 LAC::*lux*, LAC4303 (*lux*) and Xen36 (*lux*) (all 1 × 10^4^ CFU) were cultured in 96-well black polystyrene microplates with clear bottoms that were tissue culture (TC)-treated (Corning) without (none) and with the addition of different concentrations (0.03–1.2 mg/240 µL/well) of D-Luciferin (XenoLight D-Luciferin - K^+^ Salt Bioluminescent Substrate, PerkinElmer) for 0–14 hours at 240 rpm at 37 °C. Absorbance (A_600_) and bioluminescent signals (photons/0.1 second) were measured every 5 minutes on a plate reader (BioTek H1 Synergy) (n = 4 replicates with 2 iterations). All absorbance and bioluminescent signal measurements were blanked to control wells containing the same D-Luciferin concentration.

### *In vitro lux* versus *luc* BLI signals from bacterial culture plates

AH4826 (*lux* + *luc*) was cultured on TSA petri dishes (100 × 15 mm) overnight at 37 °C ± addition of 600 ng D-Luciferin (PerkinElmer) in 200 µL PBS pipetted directly onto the surface of the plates. All plates were imaged for a 30 second acquisition time (IVIS Lumina III, PerkinElmer) with no filter (open) and with 520, 570, 620, 670, 710 and 790 nm emission filters and representative images of the BLI signals are provided (n = 3 replicates with 2 iterations).

### *In vitro lux* versus *luc* BLI signal through tissue

AH4807 (*lux*), AH4775 (*luc*), AH4826 (*lux* + *luc*), USA300 LAC::*lux*, LAC4303 (*lux*) and Xen36 (*lux*) (1 × 10^9^ CFU) were cultured in 96-well black polystyrene microplates with clear bottoms that were TC-treated (Corning) without (none) and with the addition of different concentrations (0.03–2.4 mg/280 µL TSB/well) of D-Luciferin (PerkinElmer). To determine tissue penetration of the BLI signals, different thicknesses (5.25–21.0 mm) of sliced cooked ham (Lunch Mate) were placed on top of the plate covers prior to BLI. All plates were imaged for a 1 minute acquisition time at 37 °C (IVIS Lumina III) and representative images of the BLI signals are provided (n = 3 replicate wells for all conditions with 2 iterations).

### Animals (mice and rabbits)

All mouse and rabbit studies were approved by the Johns Hopkins University Animal Care and Use Committee according to the guidelines and regulations described in the Guide for the Care and Use of Laboratory Animals (National Academies Press, 2011). All mice and rabbits were maintained and housed under specific pathogen–free conditions at our animal facility accredited by the American Association for the Accreditation of Laboratory Animal Care (AAALAC) at Johns Hopkins. Six to 8 week-old female C57BL/6 mice (Jackson Laboratories, Bar Harbor, ME) were used in all *in vivo* mouse experiments. Ten to 16 week-old male Dutch Belted rabbits (Robinson Services, Mocksville, NC) (~2 kg body weight) were used in all *in vivo* rabbit experiments.

### Mouse model of *S. aureus* bacteremia

Mice were anesthetized (inhalation 2% isoflurane) and all hair on the dorsal and ventral skin was shaved with clippers. Anesthetized mice were inoculated intravenously (i.v.) with either a 20–30% lethal inoculum (1 × 10^7^ CFU) or a sub-lethal inoculum (1 × 10^6^ CFU) of LAC4303 (*lux*) or AH4826 (*lux* + *luc*) in a 100 μL volume of PBS using a 29-gauge insulin syringe via the retro-orbital vein. For the 1 × 10^7^ CFU inoculum, on day 3 post-inoculation, *in vivo* BLI was performed on anesthetized mice (2% isoflurane) from the ventral and dorsal sides of the mice before (−5 minutes) and after injection (at the indicated time points between 0 and 65 minutes) of D-Luciferin (150 mg/kg administered subcutaneously [s.c.]) for a 1 minute acquisition time at 37 °C (IVIS Lumina III). For the 1 × 10^6^ CFU inoculum, on days 1, 2 and 3 post-inoculation, *in vivo* BLI was performed on anesthetized mice (2% isoflurane) ± administration of D-Luciferin (150 mg/kg s.c. given 15–25 minutes prior to imaging) for a 1 minute acquisition time at 37 °C (IVIS Lumina III). Total flux (photons/s) was measured within an oval region of interest (2 × 4 cm) from the ventral and dorsal sides of the mice using Living Image software (PerkinElmer). To provide anatomical co-registration data, for the 1 × 10^6^ CFU inoculum, *in vivo* BLI and simultaneous CT imaging were performed on day 3 post-inoculation on anesthetized mice (2% isoflurane) ± administration of D-Luciferin (150 mg/kg s.c. given 15–25 minutes prior to imaging) for a 3 to 15 minute acquisition time (depending on signal intensity using autoexposure) at 37 °C using firefly luciferase DLIT settings (600 nm, 620 nm, 640 nm filters) on an IVIS Spectrum-CT imaging system for both *lux* and *luc* signals (PerkinElmer). To determine the *in vivo* lower limit of sensitivity and duration of the *in vivo* BLI signals of AH4775 (*luc*), 6-week old of C57BL/6 female mice were inoculated intravenously with 1 × 10^6^ CFU of AH4775 (*luc*). At 16-hours (sensitivity) or on a weekly basis up to 3-weeks post infection, mice were administered D-Luciferin (150 mg/kg s.c.) and after 20 minutes *in vivo* BLI was performed on the dorsal sides of the mice with a 5 minute acquisition time at 37 °C (IVIS Lumina III). Total flux (photons/s) from two separate oval region of interests (1.1 × 0.9 cm) overlying the right and left kidneys were measured using Living Image software (PerkinElmer). Mice were then immediately euthanized and the right and left kidneys were separately isolated, homogenized and plated overnight to enumerate the *ex vivo* CFU, as described below. The linear regression line and correlation coefficient of determination between *in vivo* BLI and *ex vivo* CFU were calculated using Prism (GraphPad, La Jolla, CA).

### Mouse model of *S. aureus* skin infection

This mouse model of *S. aureus* skin infection was performed as previously described^22^. Briefly, the dorsal backs of anesthetized mice (2% isoflurane) were shaved and injected intradermally (i.d.) in the upper back skin with 1 × 10^8^ CFU/100 μL PBS of AH4826 (*lux* + *luc*). On days 1, 3, 7 and 10, *in vivo* BLI was performed on anesthetized mice (2% isoflurane) ± administration of D-Luciferin (150 mg/kg s.c. given 15–25 minutes prior to imaging) for a 1 minute acquisition time at 37 °C (IVIS Lumina III). Total flux (photons/s) was measured within a 1 × 10^3^ pixel circular region of interest using Living Image software (PerkinElmer).

### Mouse model of a mixed *S. aureus* and *P. aeruginosa* wound infection mouse model

A mouse model of a mixed full-thickness wound infection with *S. aureus* and *P. aeruginosa* was modified from a previously established model in which the *S. aureus* inoculum was 10-fold higher than the *P. aeruginosa* inoculum^60,61^, using the *S. aureus* AH4775 (*luc*) strain and the *P. aeruginosa* (*lux*) Xen41 strain (PerkinElmer). Briefly, C57BL/6 mice were anesthetized (2% isoflurane) and the dorsal backs were shaved and the skin was surgically prepped with povidone-iodine and 70% alcohol. A full thickness 6-mm punch biopsy (Acuderm) was used to create a circular excisional full-thickness wound and AH4775 (*luc*) (2 × 10^6^ CFUs/10 µL) and Xen41 (*lux*) (2 × 10^5^ CFUs/10 µL) were sequentially pipetted into the wound bed. *In vivo* BLI of AH4775 (*luc*) and Xen41 (*lux*) was performed on anesthetized mice 20 minutes after administration of D-Luciferin (150 mg/kg s.c.) on days 1, 3, and 7 with a 1 minute acquisition time at 37 °C using 670 nm and 520 nm emission filters. These specific filters were used because the *luc* and *lux* signals from the different bacteria could be spectrally unmixed, as there was no overlap of signals. On day 7, mice were euthanized and the wound tissue was homogenized and plated overnight to enumerate the *ex vivo* CFU, as described below.

### *Ex vivo* CFU enumeration

For experiments with 1 × 10^6^ CFU of AH4826 (*lux* + *luc*), mice were euthanized on day 3 and right and left kidneys, liver and heart were harvested and homogenized (Pro200 Series homogenizer; Pro Scientific) in 1 mL of PBS at 4 °C. *Ex vivo* CFU were counted after plating serially diluted organ tissue homogenates overnight on TSA plates. For lower limit of sensitivity and duration experiments with 1 × 10^6^ CFU of AH4775 (*luc*), mice were euthanized at 16-hours and 3-weeks post-infection, respectively, and the right and left kidneys were separately harvested and homogenized (Pro200 Series homogenizer; Pro Scientific) in 1 mL of PBS at 4 °C and serially diluted homogenates were cultured overnight on TSA plates. D-Luciferin (30 mg/mL) was sprayed onto the plates and the plates were subsequently imaged in the (IVIS Lumina III) to enumerate the number of *ex vivo* CFU and to determine the percentage of CFU that still emitted a bioluminescent signal. For the mixed wound infection model with AH4775 (*luc*) and *P. aeruginosa* strain Xen41 (*lux*), mice were euthanized on day 7 post-infection and the wound tissue was excised with 8-mm punch biopsy tool (Acuderm). The excised tissue was homogenized (Pro200 Series homogenizer; Pro Scientific) in 1 mL of PBS at 4 °C and serially diluted homogenates were plated overnight on TSA plates. To distinguish between the CFU of AH4775 (*luc*) and Xen41, D-Luciferin (30 mg/mL) was sprayed onto the plates and the plates were subsequently imaged in the IVIS Lumina III using the 670 emission filter to identify and enumerate AH4775 (*luc*)-positive CFU and the 520 nm emission filter to identify Xen41 (*lux*)-positive CFU.

### Rabbit model of *S. aureus* orthopaedic implant associated infection

This model of *S. aureus* orthopaedic implant associated infection (OIAI) was performed as previously described^59^. Briefly, rabbits were anesthetized via intramuscular (i.m.) injection with ketamine and xylazine (25 mg/kg and 1.5 mg/kg respectively) (ZooPharm) and maintained with inhalation isoflurane (2%). Ophthalmic ointment (Optixcare eye lube) applied to the eyes. Sustained-release buprenorphine (ZooPharm) (0.2 mg/kg) and sustained-release meloxicam (Norbrook laboratories) (0.6 mg/dose) were given s.c. for analgesia. The right leg was shaved and ethanol and povidone-iodine (Betadine surgical scrub) (7.5%) were applied sequentially thrice. A midline incision over the patella was made followed by a medial parapatellar arthrotomy in which the patella was dislocated to visualize the femoral intercondylar notch. A 2-mm diameter hole was drilled and countersunk into the femoral medullary canal followed by bacterial inoculation with 1 × 10^4^ CFU/10 μL PBS of AH4826 (*lux* + *luc*) into the intramedullary canal. An orthopedic-grade titanium locking peg (2 × 24 mm) (Zimmer Biomet) was inserted into the femoral medullary canal so that the end was flush with the articular surface. The patella was relocated to midline and surgical site was closed in a layered fashion using 3–0 Vicryl sutures. On post-surgical days 1, 4 and 7, *in vivo* BLI was performed on anesthetized rabbits (ketamine and xylazine i.m. and 2% isoflurane) ± administration of D-Luciferin (150 mg/kg s.c. given 15–25 minutes prior to imaging) for a 5 minute acquisition time at 37 °C (IVIS Lumina III). Total flux (photons/s) was measured within a 3 × 4 cm oval region of interest using Living Image software (PerkinElmer).

### Statistics

Data for multiple comparisons were calculated by 2-way ANOVA and data for single comparisons were compared using a 2-tailed Mann-Whitney *U* test. All statistical analyses were performed using Prism (GraphPad, La Jolla, CA). Data are presented as mean ± standard error of the mean (SEM) or geometric mean and values of *P* < 0.05 were considered significant.

## Supplementary information

Supplemental Figures S1-S6

Supplemental Movie S1

Supplemental Movie S2

Supplemental Movie S3
