# Supplementary material for: Development of a Staphylococcus aureus reporter strain with click beetle red luciferase for enhanced in vivo imaging of experimental bacteremia and mixed infections
Source: Sci Rep. 2019 Nov 13;9:16663. doi: 10.1038/s41598-019-52982-0 (PMC6853927; doi:10.1038/s41598-019-52982-0)
Supplement: Supplementary file 1 — Supplemental Figures S1-S6 [file 41598_2019_52982_MOESM1_ESM.pdf]

Supplemental Information

Development of a *Staphylococcus aureus* reporter strain with click beetle red luciferase for enhanced *in vivo* imaging of experimental bacteremia

Robert J. Miller[1], Heidi A. Crosby[2], Katrin Schilcher[2], Yu Wang[1], Roger V. Ortines[1], Momina Mazhar[1], Dustin A. Dikeman[1], Bret L. Pinsker[1], Isabelle D. Brown[1], Daniel P. Joyce[1], Jeffrey Zhang[1], Nathan K. Archer[1], Haiyun Liu[1], Martin P. Alphonse[1], Julie Czupryna[3], William R. Anderson[3], Nicholas M. Bernthal[4], Lea Fortuno-Miranda[5,6] , Jeff W.M. Bulte[5,6,7,8,9], Kevin P. Francis[3,4], Alexander R. Horswill[2,10], and Lloyd S. Miller[1,11,12,13*]

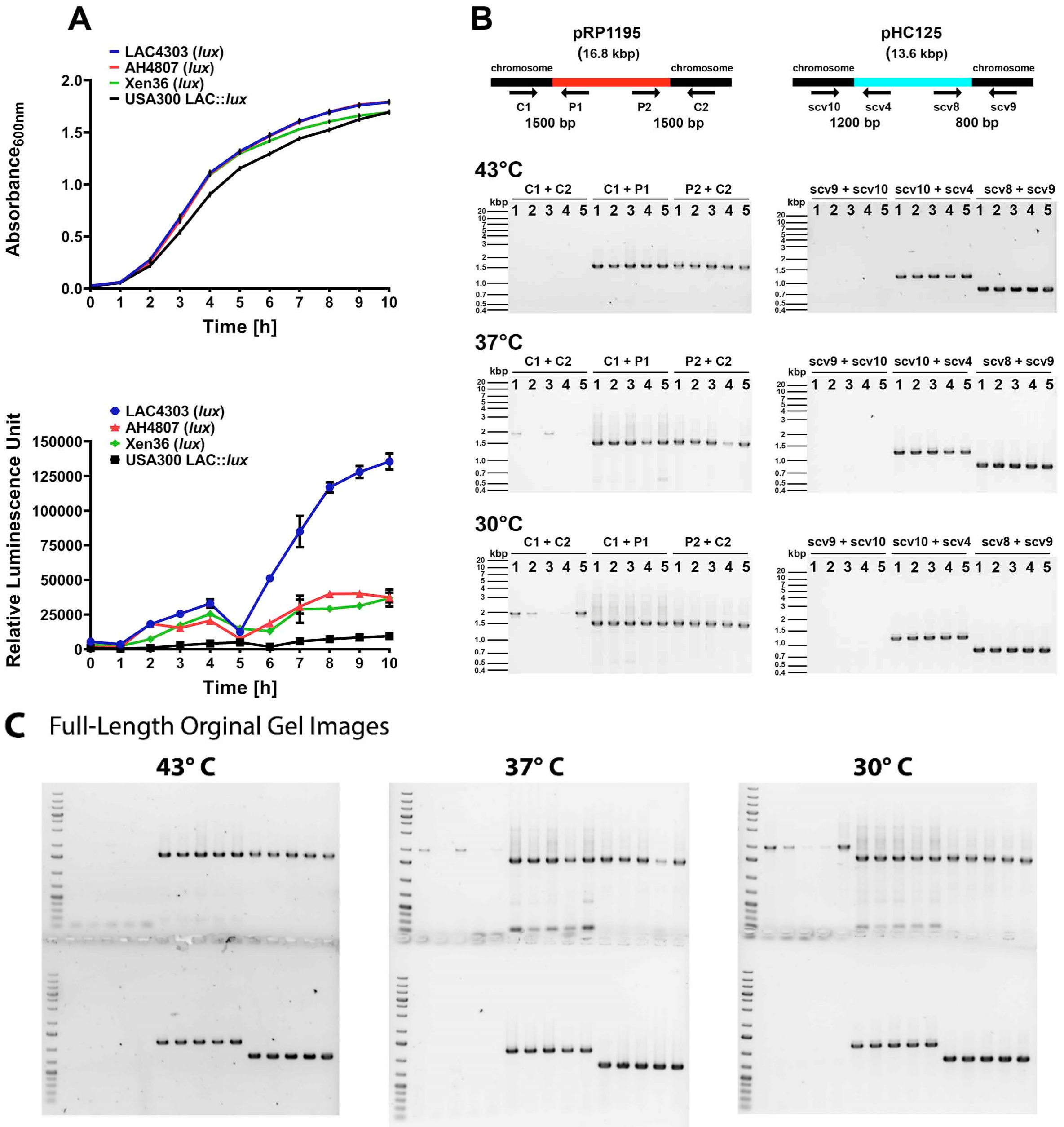

Figure S1. Comparison of the new AH4807 (*lux*) against other existing bioluminescent strains. (A) Bacterial growth (A_{600}) and relative luminescence of bioluminescent *S. aureus* strains over time. The relative luminescence units are corrected to normalized bacterial growth measured by absorbance (A_{600}). Mean values and standard deviations of three biological replicates are shown. **(B)** Location and distance between the primers used to verify the plasmid integrations. Five colonies of LAC4303 (*lux*) with integrated pRP1195 and five colonies of AH4807 (*lux*) with integrated pHC125 grown at 43°C, 37°C and 30°C were used to determine the stability of the respective integrated luminescence plasmid via PCR analysis on a 1% agarose gel. The C1 + C2 and scv10 + scv9 primer pairs allow DNA amplification (<10 kbp) in case the luminescence construct is not integrated in the chromosome. **(C)** Full-length original gel images in **(B)**.

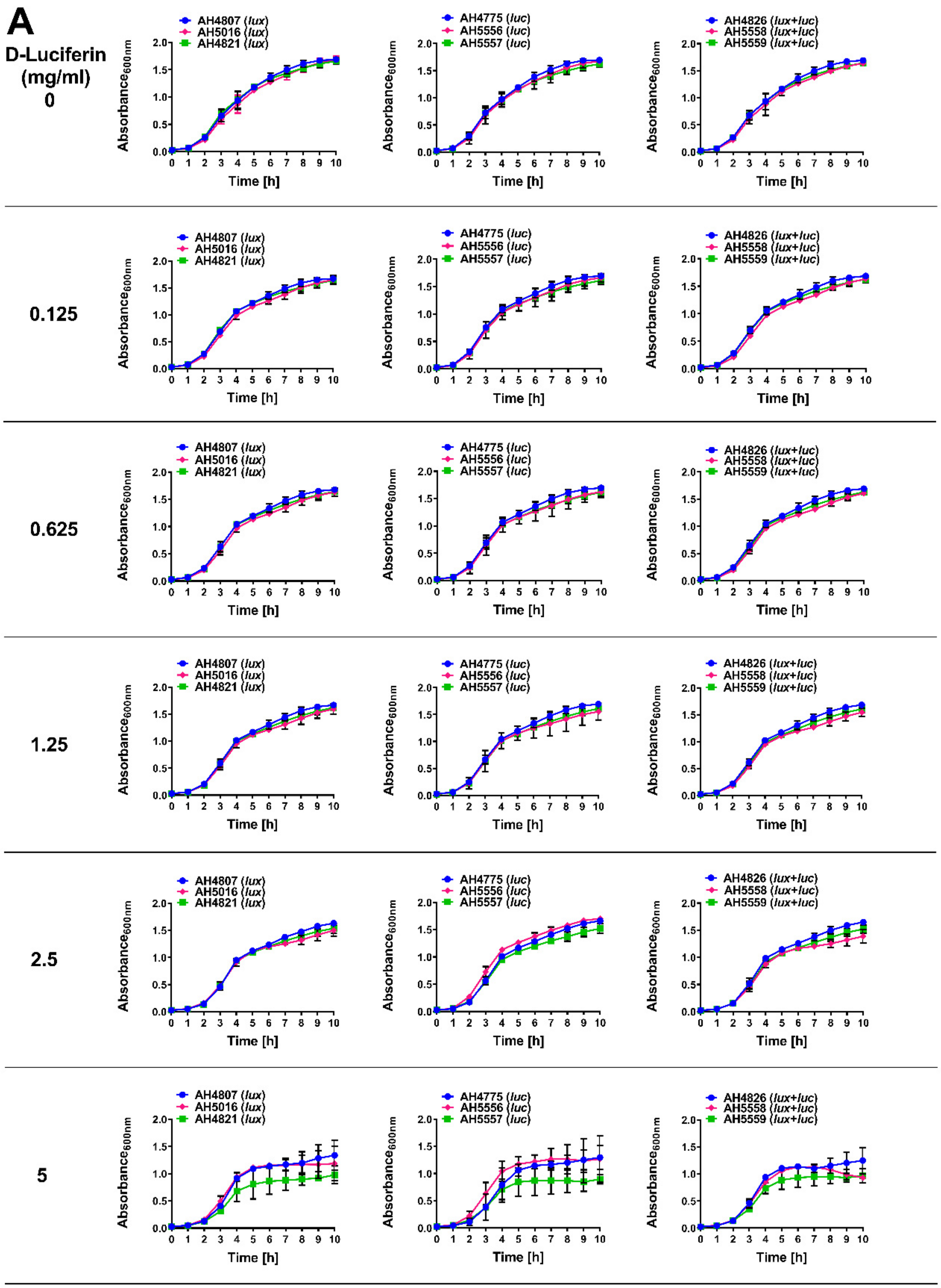
A
D-Luciferin (mg/ml)
0
0.125
0.625
1.25
2.5
5
AH4807 (lux)
AH5016 (lux)
AH4821 (lux)
AH4775 (luc)
AH5556 (luc)
AH5557 (luc)
AH4826 (lux+luc)
AH5558 (lux+luc)
AH5559 (lux+luc)
Absorbance600nm
Time [h]

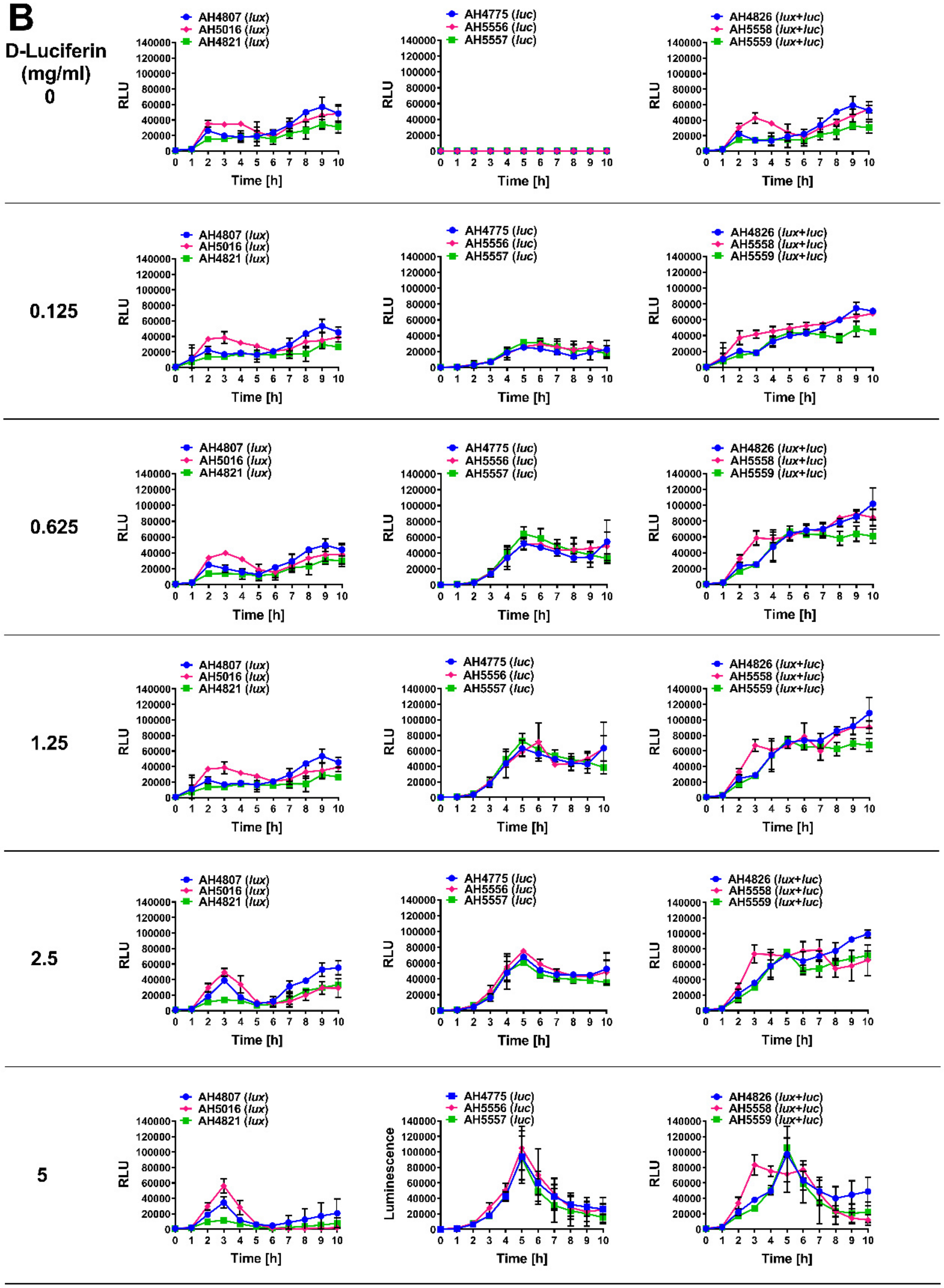

Figure S2. Comparison of bioluminescence in different *S. aureus* strain backgrounds. (A) Bacterial growth (A_{600}) and **(B)** relative luminescence units (RLU) of bioluminescent *S. aureus* strains over time without (0) or with addition of D-Luciferin (0.125 – 5 mg/ml). Blue = LAC, Pink = Newman, Green = MW2. Mean values and standard deviations of three biological replicates are shown.

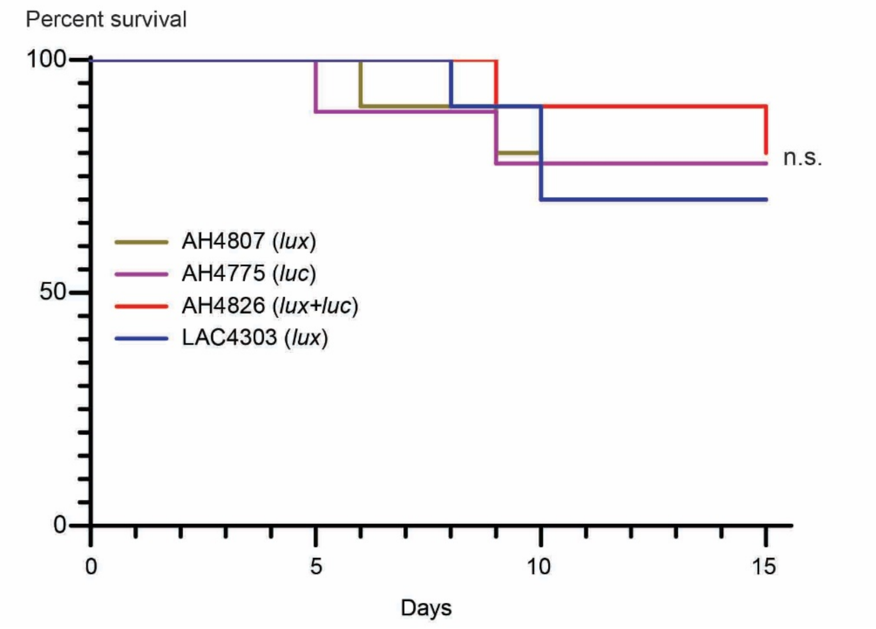

Figure S3. Percent survival of bacterial strains in the *in vivo S. aureus* bacteremia mouse model. A high inoculum (1×10^7 CFU, 20% lethal dose) of AH4807 (*lux*), AH4775 (*luc*), AH4826 (*lux*+*luc*) or LAC4303 (*lux*) was injected intravenously in mice (n=10 mice/group). Data are presented as Kaplan-Meier survival curves. Data for all bacterial strains were compared using a Log-rank (Mantel-Cox) test. No significant differences were found between the strains. n.s. = not significant.

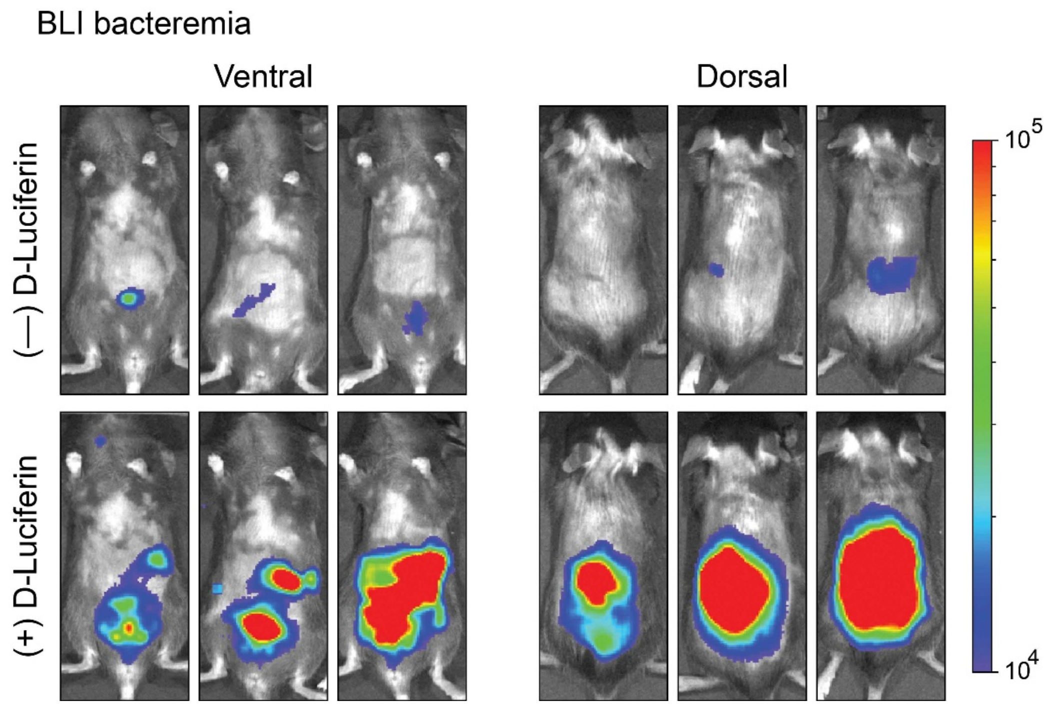

Figure S4. Additional representative *in vivo* BLI signals of *lux* versus *luc* in a *S. aureus* bacteremia mouse model shown in Figure 6A. A sub-lethal inoculum (1×10^6 CFU) of AH4826 (*lux*+*luc*) was injected intravenously in mice (n=10 mice/group) and mice were imaged ± administration of D-Luciferin (150 mg/kg s.c.) at 15-25 minutes prior to *in vivo* BLI (Fig. 6). Representative *in vivo* BLI signals of the ventral and dorsal sides of 3 mice (from 10 total mice, with similar results) on day 3 obtained using the 2D IVIS Lumina III imaging system (PerkinElmer) are shown.

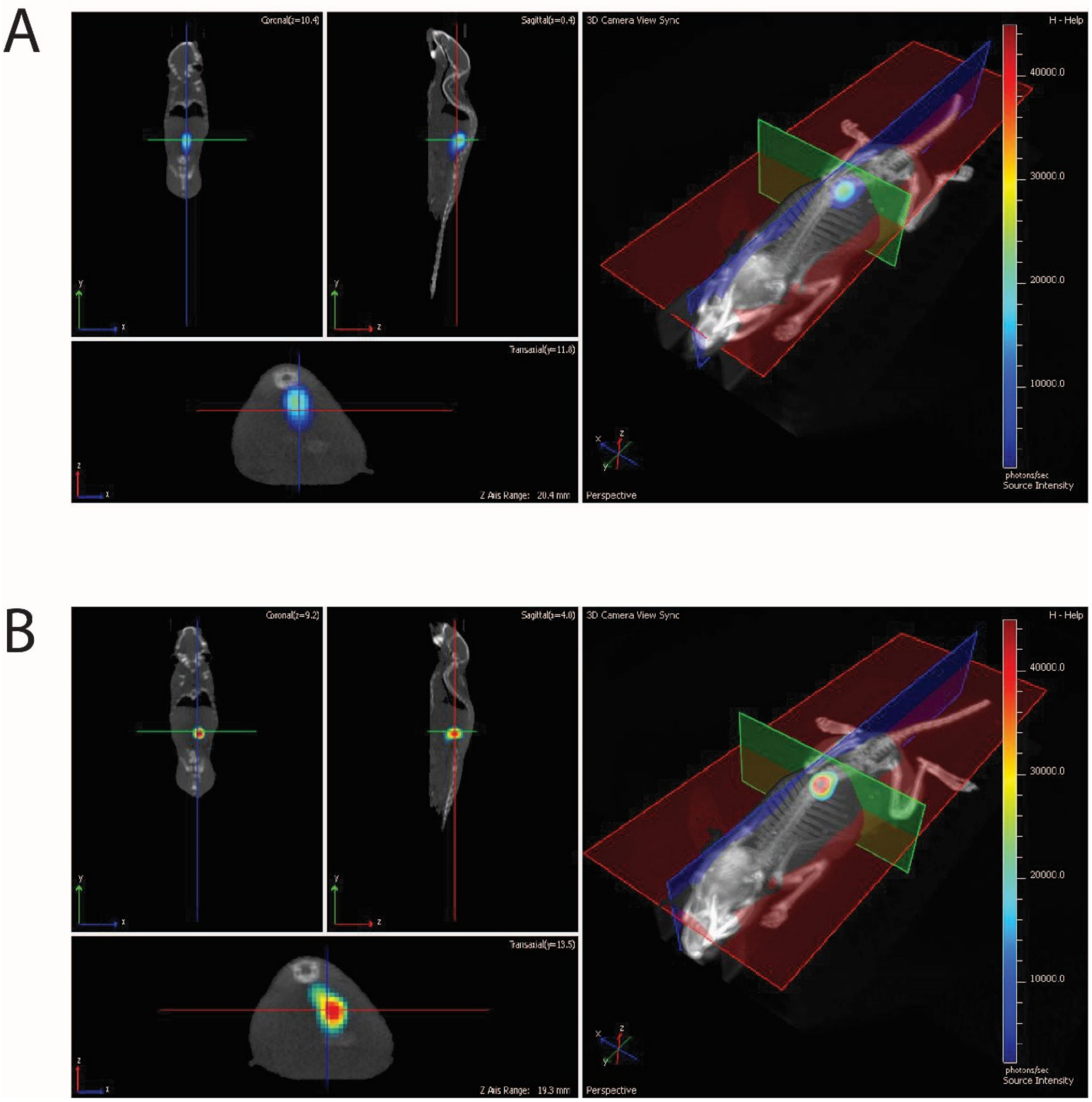

Figure S5. Additional representative *in vivo* 3D BLI signals of *lux* versus *luc* in a *S. aureus* bacteremia mouse model shown in Figure 6D. A sub-lethal inoculum ($1{\times}10^6$ CFU) of AH4826 (*lux*+*luc*) was injected intravenously in mice (n=10 mice/group) and mice were imaged ± administration of D-Luciferin (150 mg/kg s.c.) at 15-25 minutes prior to *in vivo* BLI (Fig. 6). Representative *in vivo* 3D BLI (DLIT) signals of (A) *lux* and (B) *luc* are shown for the mouse in Figure 6D. Images were acquired using a PerkinElmer IVIS Spectrum-CT and are displayed using a global color scale for visual comparison. In addition to the 3D rendering, coronal, sagittal and transaxial 2D slice views are shown with slice locations indicated by slice planes.

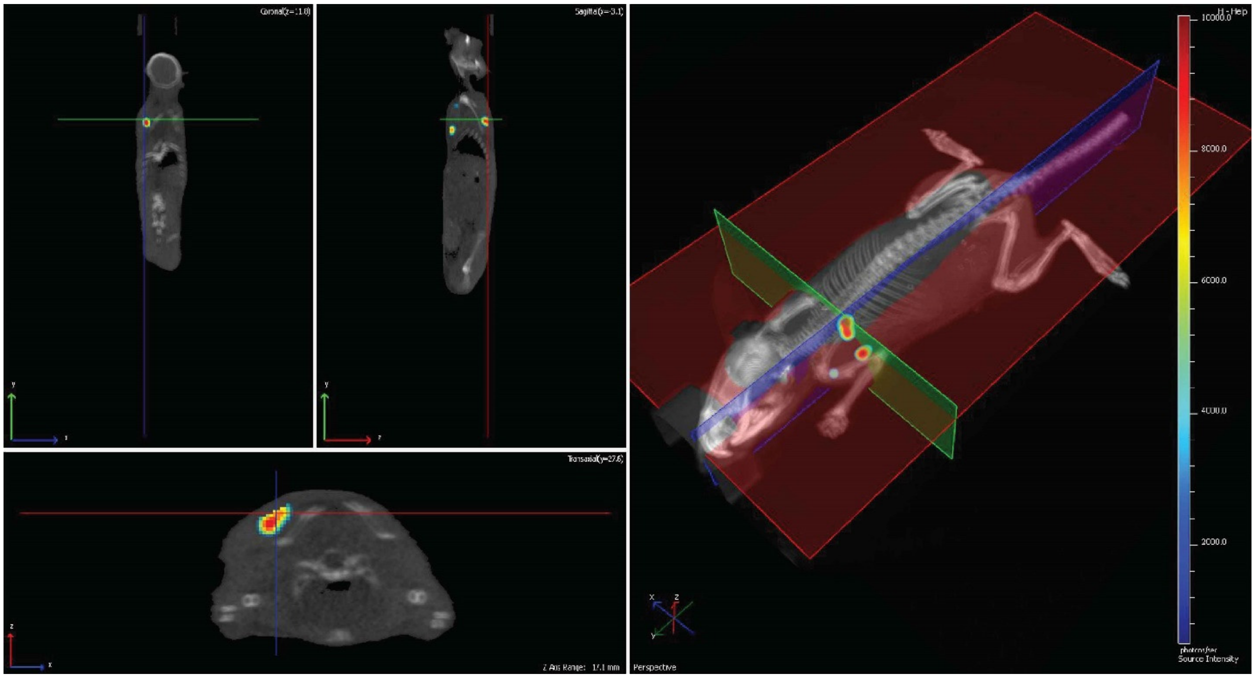

Figure S6. Additional representative *in vivo* 3D BLI signals of *luc* in a *S. aureus* bacteremia mouse model shown in Figure 6E. A sub-lethal inoculum (1×10^6 CFU) of AH4826 (*lux*+*luc*) was injected intravenously in mice (n=10 mice/group) and mice were imaged ± administration of D-Luciferin (150 mg/kg s.c.) at 15-25 minutes prior to *in vivo* BLI (Fig. 6). Representative *in vivo* 3D BLI (DLIT) image of *luc* is shown for the mouse in Figure 6E, as there was no detectable *lux* signal in this mouse. Images were acquired using a PerkinElmer IVIS Spectrum-CT. In addition to the 3D rendering, coronal, sagittal and transaxial 2D slice views are shown with slice locations indicated by slice planes.

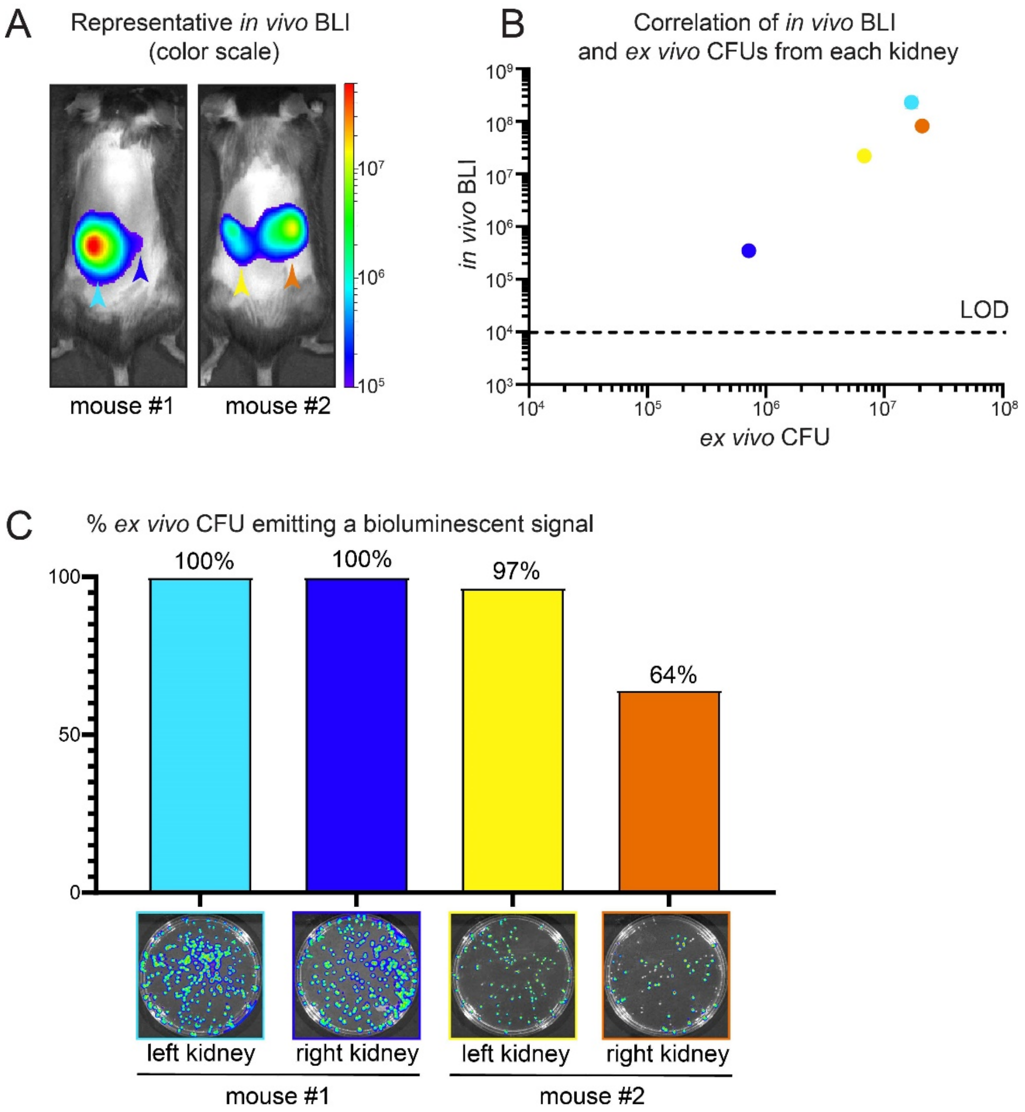

Figure S7. Duration of the detection of AH4775 (*luc*) by *in vivo* BLI in the *S. aureus* bacteremia mouse model. Three-weeks hours post-intravenous inoculation of 1×10^6 CFU of AH4775 (*luc*) in mice (n=2 mice, mouse #1 and mouse #2), D-Luciferin (150 mg/kg s.c.) was administered and after 20 minutes *in vivo* BLI signals was performed on the dorsal sides of the mice and *in vivo* BLI signals were acquired as total flux (photons/s) within region of interests corresponding to the right and left kidneys (IVIS Lumina III). Mice were then immediately euthanized and the right and left kidneys were separately isolated to determine the *ex vivo* CFU. (**A**) Representative images of *in vivo* BLI signals from the kidneys of the 2 mice imaged on the IVIS Lumina III (arrows with different colors point to the corresponding *in vivo* BLI signals). (**B**) Correlation between *in vivo* BLI (total flux [photons/s]) and *ex vivo* CFU from the right and left kidneys of the mice (different colors correspond to the right and left kidney signals in (**A**)). LOD = 1×10^4 photons/s (horizontal black dashed line). (**C**) The bacterial culture plates possessing the *ex vivo* CFU were sprayed with D-Luciferin and imaged with an open filter (IVIS Lumina III) to determine the percentage of CFU that still emitted a bioluminescent signal. Representative images of the bioluminescent signals from the plates are also shown.
